# Self-Help for Depression via E-mail: A Randomised Controlled Trial of Effects on Depression and Self-Help Behaviour

**DOI:** 10.1371/journal.pone.0066537

**Published:** 2013-06-21

**Authors:** Amy J. Morgan, Anthony F. Jorm, Andrew J. Mackinnon

**Affiliations:** 1 Orygen Youth Health Research Centre, Centre for Youth Mental Health, The University of Melbourne, Parkville, Victoria, Australia; 2 Melbourne School of Population and Global Health, The University of Melbourne, Parkville, Victoria, Australia; Linkoping University, Sweden

## Abstract

**Background:**

Self-help or self-management strategies are commonly used to deal with depression, but not all are thought to be helpful. A previous study found that sub-threshold depression symptoms were improved by an e-mail intervention that encouraged the use of evidence-based self-help strategies.

**Aim:**

To investigate whether these e-mails were effective for adults with a range of depression symptomatology including major depression.

**Method:**

The study was a parallel-group randomised controlled trial. Adult participants with any level of depressive symptoms were recruited over the internet from the United Kingdom, Australia, Canada, Ireland, New Zealand and the United States. Participants were randomised to receive a series of e-mails either promoting the use of evidence-based self-help strategies or containing depression information as a control. E-mails were sent automatically twice a week for six weeks. Depression symptoms were assessed with the self-rated Patient Health Questionnaire depression scale (PHQ-9).

**Results:**

1736 participants with a wide range of symptom severity were recruited and assigned to active (n = 862) and control (n = 874) groups. However, there was a significant attrition rate, with 66.9% lost to follow-up at post-intervention. Both groups showed large improvements in depression symptoms overall, with no significant difference in improvement at the end of the study (mean difference in improvement 0.35 points, 95% CI: −0.57 to 1.28, *d* = 0.11, 95% CI: −0.06 to 0.27), although there was a small effect at the study mid-point. Results were similar for the sub-group of participants with major depression. The active group showed small to moderate improvements in self-help behaviour (*d* = 0.40, 95% CI: 0.23 to 0.56).

**Conclusions:**

These results suggest that the e-mails were able to increase participants’ use of evidence-based self-help, but that this did not improve depression more than an attention control.

**ClinicalTrials.gov:**

NCT01399502

## Introduction

Self-help or self-management strategies are commonly used to deal with depression [1]. They are also thought to be effective, with some strategies rated as highly as professional treatments [2], [3]. A preference for managing depressive symptoms on one’s own is a key reason for the low rates of treatment seeking for depression [4]. Yet, within the general population, the use of self-help strategies is not optimal. Some commonly used strategies are known to be inefficacious and unhelpful, such as spending more time alone, drinking alcohol, and taking painkillers [5], [6]. Furthermore, use of potentially helpful strategies such as physical activity and healthy eating tends to *decrease* with depression [7], [8]. Promoting effective self-help strategies to the public as an early intervention strategy has been suggested as one way to help with the large burden of disease of depression [9]. Unlike the promotion of help seeking, this would not place a burden on stretched clinical resources. It would be analogous to health promotion campaigns on other major sources of disease burden, such as heart disease and cancer, which have raised awareness about actions that can be taken to reduce risk of disease.

Several studies have indicated that self-help advice delivered via pamphlets or letters can improve depressive symptoms in the short-term. Geisner, Neighbors and Larimer [10] targeted college students with depressive symptoms and found that mailing them feedback about their symptoms and suggesting coping strategies to try was more effective than an attention control. Similarly, García-Toro, Ibarra, Gili, Serrano, Oliván, et al. [11] gave letters to depressed outpatients recommending four self-help strategies (sleep, exercise, diet, and sunlight exposure). Despite the simplicity of the intervention, they found that depression was significantly more improved in those who received the recommendations, and data from a sub-sample indicated that exercise and sunlight exposure had increased [12]. Whilst simple to develop, letters and brochures lack some advantages of internet-based approaches, in particular e-mail, which has a wide reach, has low marginal costs per additional user, and can allow tailoring of content to the needs of individuals. It is possible to change health behaviours such as exercise and diet via e-mail messages [13]. E-mail reminders about self-help strategies have been endorsed by patients with depression as a beneficial tool [14].

To investigate whether e-mails promoting effective self-help strategies could change self-help behaviour and improve depression, we developed an e-mail-based intervention called Mood Memos. This was a fully automated system that could be easily disseminated. It delivers 12 e-mails over a 6-week period, which encourage the use of self-help strategies endorsed by experts for depression. A randomised controlled trial indicated that the e-mails were effective for improving sub-threshold levels of depression [15], and that improvement in depression was associated with more frequent usage of the promoted self-help strategies [16]. There was some indication that the Mood Memo e-mails also prevented the development of major depression, although the study was not designed or powered to detect a statistically significant lower risk from the intervention for this outcome.

Given this promising result, we were interested in whether the Mood Memos intervention would be helpful for those with full-threshold levels of depression, as the first study targeted only mild or sub-threshold depressive symptoms. Evaluating the intervention’s effectiveness in more severe levels of depression could determine whether improving self-help via e-mail is only possible or effective for mild levels of depression. It is possible that the e-mails would not be sufficiently motivating for those who are more depressed and would not change self-help behaviour. It is also possible that improving self-help behaviour would have little or no benefit and that more powerful interventions are needed for more severe depression. Therefore, the current paper reports on a second trial of the Mood Memos intervention. In contrast to the original trial, participation by individuals with any level of depressive symptoms was allowed. We also admitted participants who were already receiving depression treatment, both for ethical reasons and because it is likely that the system would often be used this way in practice. The intervention could be well suited as an adjunct to cognitive behaviour therapy, as it could reinforce lessons learned during therapy. We expected that the active Mood Memo e-mails would reduce depressive symptoms more than the control e-mails overall, as well as in sub-samples of participants with case level major depression and with sub-threshold depression. We also hypothesised that participants allocated to the active Mood Memo e-mails would increase their usage of the promoted self-help strategies, relative to participants assigned to the control e-mails.

## Methods

### Ethics Statement

This parallel-group randomised controlled trial was registered at ClinicalTrials.gov (NCT01399502; http://clinicaltrials.gov/ct2/show/NCT01399502) and was approved by the University of Melbourne Human Research Ethics Committee (see Checklist S1 for CONSORT checklist and Protocol S1 for the protocol).

### Recruitment

Participants were recruited to the study through the internet, as this allowed for broad participation, and was a successful recruitment technique in the first Mood Memos study [17]. The study was promoted mainly via paid advertising with Google, as well as through promotion by other mental health websites, online noticeboards at The University of Melbourne, and inclusion in e-mail newsletters from Mental Health First Aid Australia. Recruitment took place between November 2011 and May 2012. Paid advertising with Google was directed to individuals who had searched for a short questionnaire online to find out if they were depressed. Participants joined the study by visiting the website www.moodmemos.com. Website visitors were screened for depression symptoms with the Patient Health Questionnaire depression scale (PHQ-9) [18] and informed of their results. They were then invited to take part in the study to improve depression symptoms, by receiving e-mails with expert information or coping advice about depression. Unlike the first Mood Memos study, which was restricted to participants with sub-threshold depression, this study allowed participation by participants with any level of depression symptoms. There were few restrictions on participation, other than being aged 18 years or over, having at least weekly access to the internet, and being a resident of Australia, New Zealand, UK, Ireland, Canada or the USA. Participants already receiving treatment for depression were eligible to participate.

The effect size of the Mood Memos intervention was expected to be small, given the small effect size found in the first Mood Memos study (*d* = 0.17) and the small effect size of unsupported internet-based treatment of depression (*d* = 0.25) [19]. A power analysis indicated that a sample of 393 per condition would give 80% power to detect a small effect size (0.2 standard deviations between conditions) on a continuous outcome measure assuming a correlation of 0.5 between pre- and post-intervention scores [20]. Given the high attrition rate in the first study, we set a target of 1600 participants to allow for a 50% drop-out rate.

**Figure 1:**
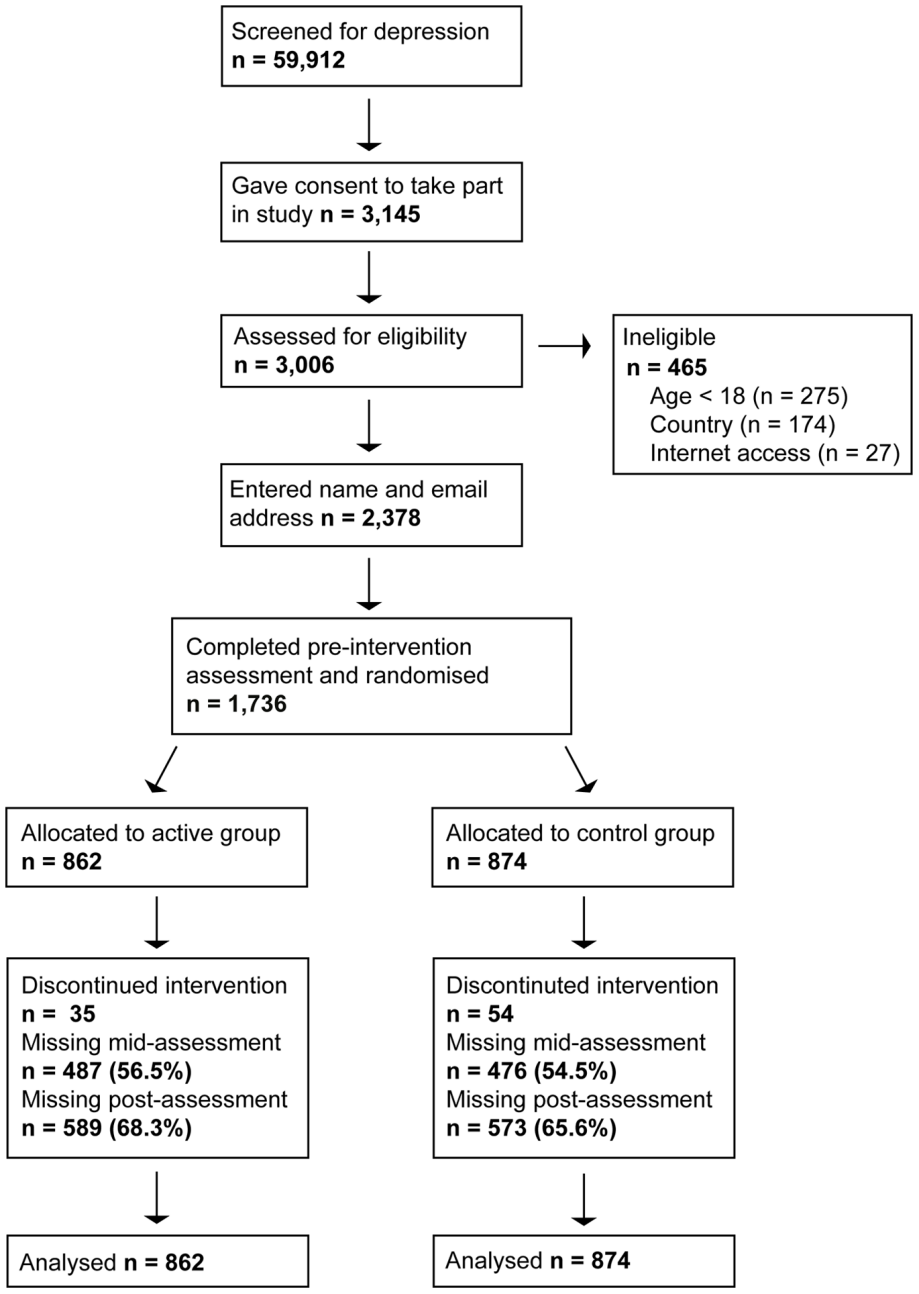
Recruitment and retention of participants.

Nearly 60,000 individuals were screened for depression symptoms on the website (see Figure 1). Of these, 3,145 (5.2%) expressed an interest in the study. Of those assessed for eligibility, 465 (15.5%) were ineligible, 2,378 entered a name and e-mail address, and 1,736 completed the pre-intervention assessment and were randomised to condition.

### Procedure

Participants were screened for eligibility, provided consent to participate by ticking a box on the website, and were asked to submit a name and e-mail address. An e-mail containing a hyperlink to the pre-intervention assessment was then sent to this e-mail address. Following the completion of the pre-intervention assessment, participants were randomised 1∶1 to the active group or control group through an automated computer script. Immediately following randomisation, participants were automatically sent their first Mood Memos e-mail.

### Interventions

The Mood Memos intervention was fully automated using PHP and a MySQL database. Participants were sent a Mood Memos e-mail twice a week for 6 weeks by the automated system, and had no interaction with a therapist. The active group received e-mails advocating self-help strategies endorsed as effective and feasible by depression experts. These e-mails were the same as used in the first Mood Memos study for sub-threshold depression. Briefly, the e-mails contained the top 14 self-help strategies that were endorsed as effective and not difficult to carry out by an international sample of depression experts who were clinicians, researchers, or consumer advocates [21]. The e-mails incorporated techniques to increase the persuasiveness of each strategy and the likelihood they would lead to behaviour change. They included a rationale for the strategy, implementation tips, solutions to barriers, how to set a goal in relation to achieving the strategy, and reminders about previous strategies. The strategies were ordered from most feasible to least feasible to implement, based on expert rankings [21]. The control group received e-mails with basic information about depression, such as its symptoms, prevalence, and risk factors, and did not suggest any action. These e-mails were designed to control for non-specific effects related to receiving e-mails with depression-related content. Screenshots of all active group e-mails are available in Active emails S1 and control group e-mails are available in Control emails S1.

### Assessment

All assessment was self-rated and undertaken on the website. Participants received up to two reminders to complete assessments a week apart, sent by automated e-mail. Assessments occurred pre-intervention, midway through the intervention (3 weeks post-baseline) and at the end of the intervention (6 weeks post-baseline).

#### Psychopathology

The primary outcome was depression symptom severity at post-intervention, assessed with the PHQ-9. The PHQ-9 assesses the frequency over the past two weeks of the nine Criterion A symptoms of DSM-IV Major Depressive Episode [22]. The PHQ-9 can be scored either using a diagnostic algorithm to make a probable diagnosis of major depressive disorder or as a continuous measure of severity. Scores range from 0 to 27 and cut-points of 5, 10, 15 and 20 represent mild, moderate, moderately severe and severe levels of depression. Secondary outcomes were psychological distress, assessed with the Kessler Psychological Distress Scale (K10) [23], and functioning, assessed with the Work and Social Adjustment Scale (WSAS) [24]. The K10 measures 10 symptoms of mental health in the anxiety-depression spectrum. Scores range from 10 (no distress) to 50 (severe distress), using the Australian scoring method [25]. The WSAS measures impairment in work, home management, social activities, private leisure activities, and ability to form and maintain relationships. Scores range from 0 to 40, with a score above 20 suggesting moderate to severe psychopathology, scores between 10 and 20 suggest significant functional impairment but less severe symptomatology, and scores below 10 are associated with subclinical populations.

#### Self-help strategy use

Self-help strategy use was assessed pre- and post-intervention only. Participants rated how frequently over the past month they had used the 14 self-help strategies promoted in the Mood Memo e-mails to improve their depressive symptoms. Frequency of use was rated on a 5-category scale (not at all, infrequently, moderately frequently, very frequently, don’t know). Ratings of mean frequency of use of each strategy were calculated, with higher figures indicating greater use (range: 0 to 3). Use of each strategy was combined into a measure of total strategy use. This total strategy use scale ranged from 0 to 42 and had good internal consistency (Cronbach’s α = .80, *N* = 1,602).

#### Adherence to intervention

Intervention adherence was measured by estimating the number of e-mails that were opened by each participant. Each e-mail contained an image located on the Mood Memos server, which allowed a count for each time the image was downloaded. This approximates whether the e-mail was read or not, but does not count participants who had images turned off in their e-mail client, or participants who received plain text versions of the e-mails instead of html e-mails.

#### Help-seeking

Participants were asked to report whether they were currently receiving treatment for depression from a health professional (e.g., doctor, counsellor, psychologist) at pre-intervention. In addition, at the mid- and post-assessment they were asked whether they had visited a health professional to help deal with depression during the previous 3 weeks. If yes, they were asked what treatments they had received, with multiple responses allowed: none, just a consultation; acceptance and commitment therapy; behaviour therapy; cognitive behaviour therapy; dialectical behaviour therapy; family therapy; mindfulness-based cognitive therapy; problem solving therapy; psychoanalytic psychotherapy; supportive counselling; psychotherapy but not sure which type; antidepressant medication; antipsychotic medication; anti-anxiety medication; mood stabilising medication; stimulant medication; medication but not sure which type. Examples of medications (i.e., brand names) were provided.

### Statistical Analysis

Analyses were undertaken on an intention-to-treat basis. Any participants who were randomised but withdrew from the study were included in the analysis as randomised. Psychopathology and self-help use outcomes were evaluated using mixed models for repeated measures (MMRM) [26]. Relationships between observations at different measurement occasions were modelled as an unstructured covariance matrix. Degrees of freedom were estimated using Satterthwaite’s approximation. Planned contrasts compared changes between groups from pre- to mid-intervention and from pre- to post-intervention. As there were significant missing data, secondary analyses were also conducted on completers-only, defined as participants that provided data pre- and post-intervention. Sub-group analyses were conducted on the primary outcome measure (the PHQ-9) in addition to analyses in the full sample. Sub-groups were defined as those meeting DSM-IV criteria for major depression at baseline, and those who had sub-threshold depression at baseline. Sub-threshold depression was defined as 2 to 4 symptoms of depression experienced more than half the days or nearly every day for two or more weeks, which have affected work, home, or social functioning. There is little consensus on how sub-threshold or sub-clinical versions of depression should best be defined [27], hence, this broad operationalization was chosen to refer to individuals who have too few symptoms to qualify for major depression, yet have a clinically relevant depressive condition. In participants with pre- and post-intervention data, reliable change on the PHQ-9 was calculated following the formula in Jacobson and Truax [28], using Cronbach’s α = .89 [29] and a standard deviation of 6.03 at pre-intervention. A change score from pre- to post-intervention greater than −5.54 indicated reliable improvement, a change greater than +5.54 indicated reliable deterioration, and change scores in between may not have reflected real change.

In the major depression sub-group, response to the intervention was compared between groups. The standard definition of clinical response on the PHQ-9 was used: a score of 10 or more at baseline, 50% improvement between pre- and post-intervention, and a post-intervention score of 9 or less [30]. Relative risk (RR), the ratio of the probability of a clinical response occurring in the active group versus the control group, was calculated and tested for significance. The number needed to treat (NNT) to achieve a clinical response was calculated with 95% confidence intervals (CI) using the method proposed by Bender [31]. A negative confidence limit indicates that the CI for the NNT runs to infinity and includes the possibility that exposure to the intervention may reduce the likelihood of protection or remission. Thus the limit of the CI is the limit of the number needed to treat (harm) (NNTH; see Altman [32]).

Several potential predictors of attrition were explored. These were pre-intervention PHQ-9 score; baseline major depression; age; gender (female); receiving treatment at baseline (yes/no); highest level of education (primary/secondary school or trade versus bachelor or postgraduate degree); history of depression (yes/no); history of bipolar disorder or psychotic disorder (yes/no); and adherence to the intervention (low, medium, high). These predictors were entered into separate logistic regression analyses with presence of data at post-intervention as the outcome variable.

Where means did not significantly differ between groups at baseline, between-group effect sizes (Cohen’s *d*) were calculated by dividing the difference between the two group means at post-intervention by their pooled standard deviation. Effect sizes based on change scores were used where there were significant baseline differences. Effects were tested at the *p*<.05 level (two-tailed), except for multiple comparisons, where the p values were adjusted using Holm’s method [33]. Analyses were carried out using IBM SPSS Statistics 20.

## Results

### Sample Characteristics

There were 1,736 participants fully enrolled in the study. Participants ranged in age from 18 to 95 years, with a mean of 37.8 years. Most participants were female, less than a third of the sample possessed at least a bachelor degree, more than half reported a history of depression, and a third reported that they were currently receiving treatment for depression from a health professional. The two groups were well matched on sociodemographic characteristics at pre-intervention (see Table 1). At pre-intervention, there were no significant differences between the control and active groups on the PHQ-9 and K10. However, the control group had a significantly higher mean score on the WSAS than the active group, *t* (1719.3) = 2.78, *p* = .006, mean difference of 1.17 (95% CI: 0.34 to 2.00). Scores on the PHQ-9 at pre-intervention ranged from 0 to 27, but on average indicated moderately severe depression (*M* = 16.7, *SD* = 5.87). The median number of symptoms experienced was 6 (range 0 to 9). Two-thirds of participants (66.0%) met criteria for a major depressive episode, this proportion was not significantly different between groups, χ^2^ (1, *N* = 1736) = 2.81, *p* = .094. Mean scores on the K10 indicated a very high level of psychological distress [34]. Mean scores on the WSAS at pre-intervention indicated moderately severe functional impairment.

**Table 1:** Pre-intervention characteristics of the total sample.

Outcome	Active (*n* = 862)	Control (*n* = 874)	Total (*N* = 1736)
Age *M* (*SD*)	37.7 (13.7)	38.0 (13.8)	37.8 (13.8)
Female (%)	79.4	79.4	79.4
Highest education level %			
Postgraduate degree	10.7	9.6	10.1
Bachelor degree	18.8	18.4	18.6
Trade-vocational diploma or certificate	31.7	34.1	32.9
Secondary/high school or less	35.5	35.5	35.5
Primary/elementary school or less	3.4	2.4	2.9
Country of residence %			
Australia	20.2	18.2	19.2
United Kingdom	53.8	53.7	53.7
Canada	11.6	11.4	11.5
United States	3.7	4.0	3.9
Ireland	6.8	8.6	7.7
New Zealand	3.8	4.1	4.0
History of depression %	57.9	55.6	56.7
History of bipolar disorder or psychotic disorder %	4.8	3.6	4.2
Currently receiving depression treatment %	32.5	34.8	33.6

Participants were free to seek treatment for depression during the study. Of those who provided data at mid- or post-intervention, 43.6% (366/839) reported receiving treatment prior to the mid- or post-assessment. Overall, more than half (55.9%, 469/839) of these participants reported receiving treatment for depression at some point during the study (including pre-intervention). About a third of participants (267/839, control group: 32.2%, active group: 31.4%) reported receiving some form of medication, primarily antidepressants, during the study. A quarter of participants (215/839) reported receiving some form of psychotherapy during the study (control group: 26.9%, active group: 24.3%).

### Attrition

A minority of participants elected to stop receiving the intervention (n = 94, 5.4%), with slightly more withdrawing from the control group than the active group (6.2% versus 4.1%), χ^2^ (1) = 4.00, *p* = .045. There was large attrition from assessments at mid- and post-intervention (see Figure 1). The attrition rate at the mid-intervention assessment was 55.5%, which increased to 66.9% at the post-intervention assessment. The attrition rate at post-intervention was not significantly different between groups χ^2^ (1) = 1.50, *p* = .220. Reminders to complete assessments had a small effect on attrition. Of participants with post-intervention data, 64% completed assessments with no reminders, 21% completed after receiving one reminder, and 15% completed after receiving two reminders. Participants with data at post-intervention were significantly more likely to: have a history of depression OR = 1.73 (95% CI: 1.40 to 2.13) *p*<.001, have a history of bipolar disorder or psychotic disorder OR = 1.68 (95% CI: 1.05 to 2.70) *p* = .031, have a university education OR = 1.87 (95% CI: 1.51 to 2.33) *p*<.001, be older OR = 1.03 (95% CI: 1.02 to 1.03) *p*<.001, report being in treatment for depression at the beginning of the study OR = 1.96 (95% CI: 1.59 to 2.41) *p*<.001, and have read more than 4 of the intervention e-mails (5 to 8 e-mails, OR = 2.95 (95% CI: 2.09 to 4.15) *p*<.001; 9 to 12 e-mails, OR = 8.57 (95% CI: 6.41 to 11.5) *p*<.001). Although pre-intervention PHQ-9 score was not a significant predictor OR = 0.98 (95% CI: 0.97 to 1.00) *p* = .086, participants who met criteria for major depression were *less* likely to have data at post-intervention OR = 0.80 (95% CI: 0.65 to 0.98) *p* = .035. Gender was not a significant predictor, OR = 0.99 (95% CI: 0.77 to 1.27) *p* = .937.

### Psychopathology Outcomes

#### Overall

**Figure 2:**
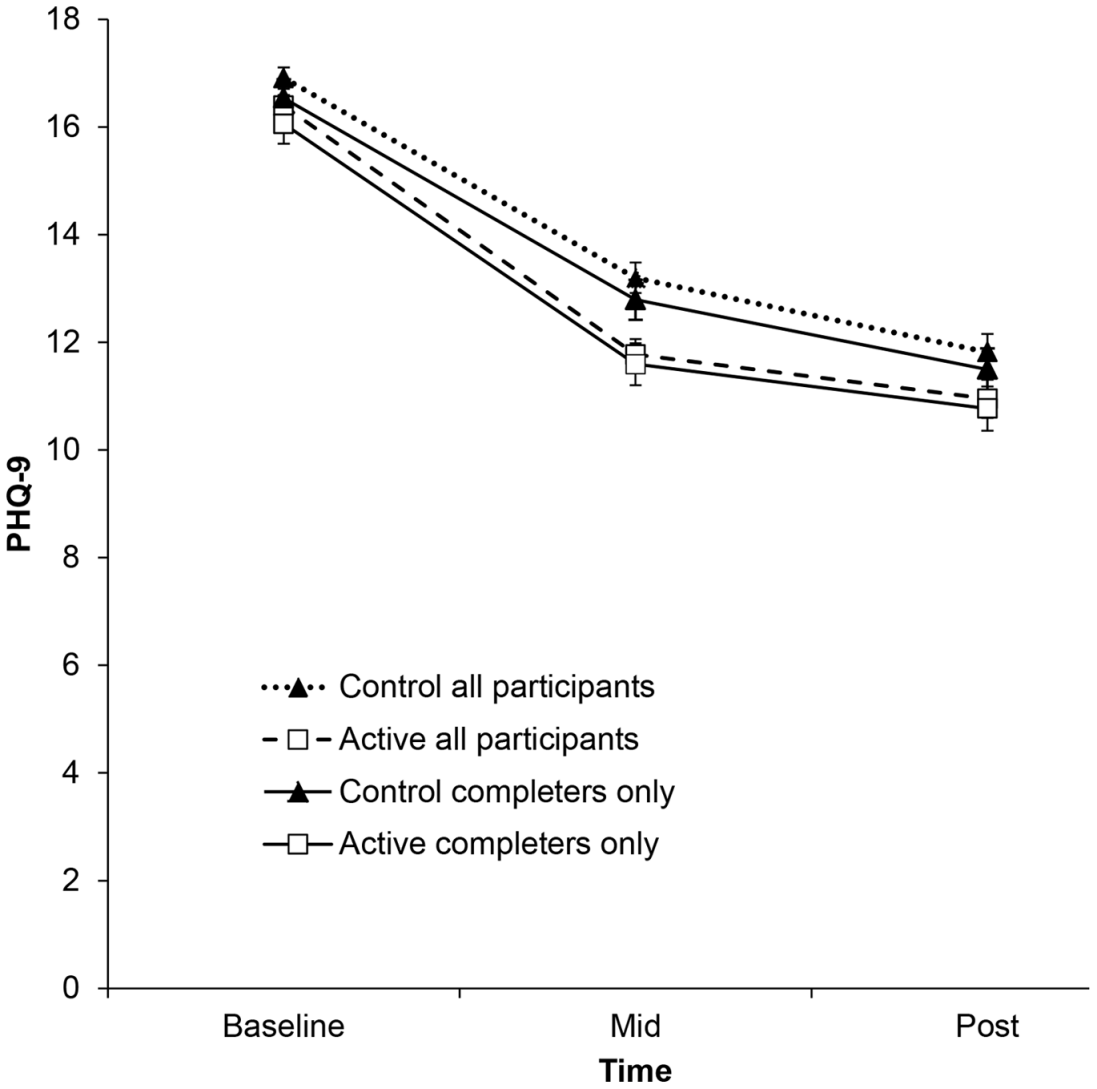
Estimated marginal means and standard errors for PHQ-9 scores, estimated under group-by-time model.

The means and standard deviations of the primary and secondary outcomes at pre-, mid-, and post-intervention are shown in Table 2. The interaction of group and time was significant for the PHQ-9, *F* (2, 693.1) = 3.14, *p* = .044 (see Figure 2). However, planned contrasts showed that this difference was primarily accounted for by the difference in improvement between groups from pre- to mid-intervention, *t* (867.1) = 2.44, *p* = .015. Mean improvement in the active group was 0.91 points (95% CI: 0.18 to 1.65) more than the control group, *d* = 0.23 (95% CI: 0.09 to 0.37). The mean difference in improvement between pre- and post-intervention was not significant, *t* (692.4) = 0.75, *p = *.453, mean difference in improvement 0.35 points (95% CI: −0.57 to 1.28), *d* = 0.11 (95% CI: −0.06 to 0.27). A similar pattern of results was found for the K10. The interaction between group and time on the K10 was significant, *F* (2, 677.2) = 3.94, *p* = .020, but differences between groups in change from pre-intervention to post-intervention were not significant, *t* (663.9) = 0.84, *p = *.399. However, the active group improved significantly more than the control group between pre- and mid-intervention, *t* (817.1) = 2.77, *p* = .006, mean difference in improvement 1.2 points (95% CI: 0.36 to 2.11), *d* = 0.22 (95% CI: 0.08 to 0.36). The interaction between group and time for the WSAS was not significant, *F* (2, 676.9) = 2.84, *p* = .059, and there were also no significant differences between groups on the change from pre- to post-intervention, *t* (675.7) = 0.76, *p* = .445, *d* = 0.03 (95% CI: −0.13 to 0.20). Again, the active group showed more improvement than the control group from pre- to mid-intervention, *t* (833.7) = 2.33, *p* = .020, *d = *0.12 (95% CI: −0.02 to 0.26). Data on adverse events were not collected in this study, however, a small proportion of participants (3.0%) recorded an individually reliable deterioration in PHQ-9 scores from pre- to post-intervention (control group: 4.0%, active group: 1.8%).

**Table 2:** Observed means, standard deviations, and sample size for psychopathology outcome measures for each group at pre-, mid-, and post-intervention for all participants and completers only.

	All participants						Completers only					
Outcome	Control			Active			Control			Active		
*PHQ9*	M	SD	n	M	SD	n	M	SD	n	M	SD	n
Pre-intervention	16.9	5.76	874	16.4	5.98	862	16.5	6.04	301	16.1	6.02	273
Mid-intervention	13.1	6.53	398	11.7	6.21	375	12.7	6.19	264	11.7	6.37	244
Post-intervention	11.5	6.72	301	10.8	6.84	273	11.5	6.72	301	10.8	6.84	273
*K10*												
Pre-intervention	33.1	7.46	873	32.5	8.01	862	32.0	7.62	301	31.8	8.14	273
Mid-intervention	28.8	8.69	398	26.9	8.98	375	28.3	8.41	264	26.9	9.16	244
Post-intervention	26.0	8.98	301	25.2	9.75	273	26.0	8.98	301	25.2	9.75	273
*WSAS*												
Pre-intervention	26.4	8.44	873	25.2	9.11	862	26.3	8.76	301	24.4	9.27	273
Mid-intervention	23.7	9.46	398	20.9	10.4	375	23.4	9.34	264	20.5	10.4	244
Post-intervention	21.2	10.6	301	19.0	11.2	273	21.2	10.6	301	19.0	11.2	273

#### Completers only

Similar results were obtained when analyses were restricted to participants that provided complete data pre- and post-intervention (n = 574; see Table 2). The interaction of group and time was not significant for the PHQ-9, *F* (2, 545.5) = 1.42, *p* = .243 (see Figure 2) and the mean difference in improvement between pre- and post-intervention was not significant *t* (572.0) = 0.48, *p* = .634. The interaction between group and time on the K10 was not significant, *F* (2, 546.1) = 2.71, *p* = .068, and differences between groups in change from pre-intervention to post-intervention were also not significant, *t* (572.0) = 0.89, *p* = .375. Lastly, the interaction between group and time for the WSAS was not significant, *F* (2, 544.1) = 1.36, *p* = .258, and there were no significant differences between groups on the change from pre- to post-intervention, *t* (572.0) = 0.40, *p* = .692.

#### Major depression

There was a similar pattern of results on the PHQ-9 when the analysis was restricted to the 1,145 participants who met diagnostic criteria for major depression at baseline (*M* = 19.9, *SD* = 3.94). The MMRM interaction of group and time narrowly escaped significance, *F* (2, 422.2) = 2.89, *p* = .057, the mean difference in improvement between pre- and post-intervention was not significant, *t* (410.4) = 0.95, *p = *.345, but the contrast between pre- and mid-intervention was significant, *t* (527.4) = 2.39, *p* = .017. The mean difference in improvement from pre- to mid-intervention was 1.19 points (95% CI: 0.21 to 2.17), *d* = 0.16 (95% CI: −0.01 to 0.34), but this was much smaller and non-significant between pre- and post-intervention, 0.60 points (95% CI: −0.65 to 1.85), *d* = 0.06 (95% CI: −0.15 to 0.27). Overall, mean PHQ-9 scores post-intervention were much reduced but still within clinical range (*M = *13.1, *SD* = 6.86). A larger proportion of participants in the active group (n = 52, 32.1%) achieved a clinical response than the control group (n = 53, 26.9%), but the intervention response rate was not significantly different (RR = 1.19, 95% CI: 0.87 to 1.64). The number needed to treat to achieve one clinical response to the intervention was 19.3 (95% CI: 6.8 to ∞ to NNTH 23.8).

#### Sub-threshold depression

A sub-group analysis was also conducted on participants who met criteria for sub-threshold depression at baseline (n = 396), to explore whether the first Mood Memos study results were replicated. Mean PHQ-9 scores at baseline were slightly but significantly higher in the control group (*M* = 11.1, *SD* = 2.17) than in the active group (*M = *10.7, *SD* = 2.02), *t* (394) = 2.17, *p* = .031. The MMRM interaction of group and time was not significant, *F* (2, 160.2) = 0.50, *p* = .609. Mean difference in improvement between groups was not significantly different between pre- and post-intervention, *t* (156.5) = 0.95, *p* = .345, 0.47 points (95% CI: −1.10 to 2.04), *d = *0.08 (95% CI: −0.25 to 0.41); or between pre- and mid-intervention *t* (191.0) = 0.99, *p* = .326, 0.6 points (95% CI: −0.60 to 1.81), *d* = 0.17 (95% CI: −0.12 to 0.46).

### Self-help Usage Outcomes

At pre-intervention there was no significant difference on the strategy use scale between the active group (*M* = 15.1, *SD* = 7.2,) and control group (*M* = 14.6, *SD* = 7.3), *t* (1,734) = −1.35, *p* = .177. An MMRM indicated significant differences over time between groups, *F* (1, 617.0) = 22.6, *p*<.001. The active group improved by a mean of 2.63 points more than the control group (95% CI: 1.54 to 3.72) on the strategy use scale, *d* = 0.40 (95% CI: 0.23 to 0.56). Results from completers only were very similar, with significant differences over time between groups, *F* (1, 572.0) = 20.2, *p*<.001, and a mean difference in improvement of 2.62 points (95% CI: 1.48 to 3.77), *d* = 0.40 (95% CI: 0.23 to 0.56). In addition to evaluating the effect of the intervention on overall usage of the promoted self-help strategies, the effect on individual strategies was explored to examine whether the e-mails had more effect on some strategies than others. Table 3 presents the results of the group by time interaction from the MMRM conducted on each self-help strategy. Eight out of fourteen strategies showed significant differences in use over time between the two groups, and all changes were in the direction favouring the active group. However, after adjusting for multiple comparisons, there were only four strategies that were used significantly more frequently by participants in the active group compared to the control group. These differences in frequency of use were small to medium in size (*d* = 0.27 to *d* = 0.47).

**Table 3:** Differences between groups in frequency of use of self-help strategies over time.

E-mail order,% read^a^	Strategy	F-ratio^b^(group bytime)	*p*	Effect size (*d*)(95% CI)
1 (89.9)	You made sure you got out of the house for at least a short time each day	15.81	<.001^c^	0.47 (0.31 to 0.64)
2 (73.4)	You tried to remain involved in purposeful activities for at least a small partof every day	10.80	.001^c^	0.27 (0.11 to 0.44)
3 (64.2)	You rewarded yourself for reaching a small goal	19.36	<.001^c^	0.43 (0.26 to 0.59)
4 (59.1)	You ate a healthy, balanced diet	4.64	.032	0.23 (0.07 to 0.40)
5 (56.7)	You made sure you got enough sleep at night and had a bed time and risingtime that varied little from day to day	3.12	.078	0.19 (0.02 to 0.35)
5 (56.7)	You tried methods to improve your sleep	2.56	.110	0.09 (−0.08 to 0.25)
6 (54.5)	You did something you enjoy	2.59	.108	0.18 (0.02 to 0.35)
6 (54.5)	You engaged in an activity that gave you a feeling of achievement	2.74	.098	0.24 (0.08 to 0.41)
7 (52.9)	You talked over problems or feelings with someone who is supportive and caring	2.61	.107	0.14 (−0.02 to 0.31)
8 (47.1)	You engaged in exercise or physical activity	5.47	.020	0.26 (0.09 to 0.42)
9 (47.8)	You made a list of strategies that have worked in the past for depression andused them	12.79	<.001^c^	0.31 (0.14 to 0.47)
10 (45.2)	You let family and friends know how you are feeling so that they are aware of whatyou are going through	3.90	.049	0.10 (−0.06 to 0.27)
11 (44.4)	You enlisted a trusted friend or relative to help you get out and about ordo activities	3.70	.055	0.17 (0.00 to 0.33)
12 (44.4)	You learnt relaxation methods	5.90	.015	0.17 (0.00 to 0.33)

a Percentage of active group participants who read any e-mail.

b Denominator degrees of freedom varied between 638 and 697.

c Estimate remained statistically significant after adjusting for multiple comparisons.

### Adherence

Both groups had a similar pattern of e-mail views. There was a sharp drop in the number of views between the first and second e-mail (81.6% of the active group, 80.0% of the control group) and then a low rate of attrition for the remaining e-mails, with the twelfth e-mail viewed by 49.3% of the active group, and 49.9% of the control group. However, participants showed a mixed pattern of e-mail views, with some e-mails missed but later ones viewed. The mean number of e-mails viewed in the entire sample was 6.8 (*SD* = 4.0). However, the distribution was U-shaped, so the mean does not accurately summarise the distribution. The number of e-mail views was split into a 3-level ordinal variable: viewers of few e-mails (1 to 4), viewers of a medium number of e-mails (5 to 8), and viewers of most e-mails (9 to 12). Approximately two-fifths of participants (37.6%) read four or fewer e-mails, one fifth (20.6%) read 5 to 8 e-mails, and two-fifths (41.8%) read 9 or more e-mails. The proportion of participants in these categories did not significantly differ between groups, χ^2^ (2) = 1.16, *p = *.559. To explore the effect of adherence on depression outcomes, a linear regression was conducted using e-mail views categorised into three levels. Incremental (linear) and non-linear effects of email view category were evaluated. Adjusting for pre-intervention depression score and group allocation, e-mail views was a significant predictor of post-intervention depression score, *B* = −0.71 (95% CI: −1.38 to −0.04), *p* = .038, but the percentage of variance accounted for was very small when it was added to the model (ΔR^2^ = .006). The addition of a quadratic e-mail views term was almost significant *B* = 0.43 (95% CI: −0.02 to 0.87), *p* = .062, ΔR^2^ = .005. Adherence was also examined to see whether it was associated with clinical response in participants with major depression at baseline. In the active group, intervention response rates were 25.0%, 35.3% and 36.4% for those who read 1 to 4 e-mails, 5 to 8 e-mails, and 9 to 12 e-mails, respectively. This suggests some benefit from reading more than 4 e-mails, but little additional benefit from reading 9 or more. Whereas in the control group, response rates were 25.0%, 36.1% and 21.1%, for those who read 1 to 4 e-mails, 5 to 8 e-mails, and 9 to 12 e-mails, respectively.

## Discussion

This randomised controlled trial failed to show a benefit for the Mood Memo e-mails relative to an attention control on depression, psychological distress or psychological functioning. Both groups showed large improvements over the course of the intervention, with little difference in improvement between them by the end of the study. Although there was a consistent pattern of a small effect at the mid-point of the study, this had faded by the end of the intervention. Results from the sub-sample of participants with sub-threshold depression did not replicate the effects on depression found in the first Mood Memos study, as the post-intervention effect size was much smaller [15]. However, the number of participants with sub-threshold depression meant the study was underpowered to detect a statistically significant difference in mean improvement and the confidence intervals were broad, encompassing the effect observed in the first study. In participants with case-level depression at baseline, there was a small, non-significant difference in intervention response rates between groups.

Despite the negligible effect on psychopathology outcomes, the e-mails did have a small-to-moderate effect on self-help behaviour. The active e-mails increased use of the promoted self-help strategies overall, but particularly for the strategies in the first three e-mails: you made sure you got out of the house for at least a short time each day, you tried to remain involved in purposeful activities for at least a small part of every day, and you rewarded yourself for reaching a small goal. This would suggest that the null result on depression was not because the e-mails were unsuccessful in changing self-help behaviour. Rather, these behaviour changes were insufficient, over and above background treatments and placebo effects, to improve depression. A significant proportion of participants in both groups received a professional treatment for depression (e.g., antidepressants or psychotherapy), and perhaps the anticipated small effect of the intervention on depression was overwhelmed by the larger benefits of depression treatments. It is also possible that the increases in self-help behaviours were too small, and that larger changes could be achieved by modifying the intervention. Some potential modifications include tailoring on dose, use of strategies, and stage of change for managing depression [35], as well as enhancing the capacity to set and track goals related to self-help.

A sub-group of participants were highly adherent to the intervention, reading at least 75% of the e-mails, but there was also a significant proportion who read four or fewer e-mails. Adherence in web-based depression self-help interventions is often measured in number of modules completed, and evidence suggests a positive relationship between adherence and outcomes [36]. Yet it is also true that, given the ease of discontinuing from an e-health intervention, participants who improve early may drop-out rather than continue to use the intervention when they believe it is no longer required [37]. The current study found only a very small relationship between adherence and depression outcome. Although the e-mail strategies were all thought to be helpful at a population level, not all strategies may have been helpful or necessary at an individual level. Hence, one person may have found engaging in the strategies promoted in the first few e-mails to be helpful, and therefore did not open the remaining e-mails. Conversely, another person may have found the e-mails of minimal help, but continued to open subsequent e-mails hoping to find a benefit. For these reasons, use of the self-help strategies promoted in the e-mails may be a better indicator of outcome than e-mail views.

The finding that e-mail messages that promoted self-help behaviours for depression led to increases in those behaviours is encouraging. These results support other research showing periodic prompts and reminders to engage in health behaviours can be effective in improving health outcomes, at least in the short term [38]. Although these behaviours did not have a significant impact on depression, the results have implications for managing chronic diseases and improving physical health. Interventions delivered by mobile phone, including text messaging and smartphone applications, are showing promising results in areas such as diabetes management, smoking cessation, weight loss and increasing physical activity [39], [40]. These interventions have the potential to deliver timely prompts to engage in health-promoting behaviours and could have public health impact with their scalability and low marginal costs per additional user.

Assessment attrition was substantial and associated with clinical history, level of education, intervention adherence and case-level depression at baseline. The e-mail reminders reduced attrition somewhat, but the high rate of attrition was not unexpected given the lack of participant supervision and ease of ignoring e-mail requests to complete assessments. The data were analysed with mixed models for repeated measures, which retains all available data from participants rather than deleting cases with incomplete data or replacing missing data with the last valid observation. These analyses were consistent with the results of completers only. The study was also limited in its reliance on self-report measures to assess depression and self-help behaviour. A clinical interview may have identified individuals with depression caused by a medication or medical condition, which would not have been amenable to intervention with self-help. Clinician-rated scales are also considered the gold standard in depression outcome research. Recent research suggests however that both clinician-rated and self-rated instruments should be used, because they each provide unique information and their estimates of treatment efficacy differ significantly [41], [42]. Nevertheless, the method of recruiting participants using online self-rated screening with few exclusionary criteria was a naturalistic approach with good external validity. The intervention reached people who were not already receiving treatment and recruited an international sample of participants including individuals with lower educational attainment, a group often underrepresented in intervention uptake and research.

Overall, the effects on psychopathology must moderate the encouraging results found in the first study. This is disappointing because the intervention required minimal resources to operate and could have been widely disseminated if it proved effective. Other, admittedly more intensive, internet-based interventions for depression are available and have been shown to be effective in several meta-analyses [19], [43], particularly when used under professional guidance. Although the present intervention did not work under unguided conditions, it remains possible that it may have some effect under guided conditions.

## Supporting Information

Checklist S1 **CONSORT checklist.**
(DOC)

Protocol S1 **Trial protocol.**
(PDF)

Active emails S1 **Screenshots of the active group emails.**
(PDF)

Control emails S1 **Screenshots of the control group emails.**
(PDF)
