# Supplementary material for: Self-Help for Depression via E-mail: A Randomised Controlled Trial of Effects on Depression and Self-Help Behaviour
Source: PLoS One. 2013 Jun 21;8(6):e66537. doi: 10.1371/journal.pone.0066537 (PMC3689826; doi:10.1371/journal.pone.0066537)
Supplement: Protocol S1 — Trial protocol. (PDF) [file pone.0066537.s002.pdf]

FORM 1

THE UNIVERSITY OF MELBOURNE
HUMAN RESEARCH ETHICS COMMITTEE

APPLICATION FOR APPROVAL OF A PROJECT INVOLVING HUMAN PARTICIPANTS

PROJECT REFERENCE DETAILS

**Enter the Ethics ID number assigned by Themis Research to this ethics application.**	0931313.1
**Enter the title of the Project as recorded in Themis Research**	Promotion of self-help strategies for sub-threshold depression via the internet: Randomised controlled trial
**Enter the name of the Responsible Researcher as recorded in Themis Research**	Prof Anthony Jorm

1. PROJECT DETAILS

1.1 **EXECUTIVE SUMMARY IN PLAIN ENGLISH:** *Provide a brief summary of the project outlining the broad aims, background, key questions, research design/approach, the participants in the study and what they will be asked to do, and the importance or relevance of the project.* [This description must be in everyday language, free from jargon, technical terms or discipline-specific phrases. (No more than 300 words).]

People who are not clinically depressed, often have some depressive symptoms. These symptoms impair the person's functioning and may develop into major depression. Self-help strategies, such as exercise or taking time off work, are acceptable and often used by those with depressive symptoms. However, some strategies are not as effective as others, and some may be harmful (e.g. substance use). Previous research has identified a set of self-help strategies thought to be helpful by an international group of depression experts. This project will test the effectiveness and feasibility of promoting these helpful self-help strategies to individuals with depressive symptoms (but not major depression) in a randomised controlled trial. The internet will be used to recruit participants, assess outcomes and deliver the intervention, which will consist of email messages based on the self-help strategies. The effect of these will be compared to receiving emails containing general information about depression. A variety of outcomes will be assessed, including effects on symptoms, general wellbeing, and social and occupational functioning. The aims are twofold: (1) to assess the feasibility of conducting a health promotion campaign of self-help messages for individuals with some depression symptoms, and the viability of using the internet for the campaign; and (2) to reduce the overall level of depressive symptoms as well as the number of individuals developing major depression.

1.2 **AIMS OF AND JUSTIFICATION FOR THE RESEARCH:** *State the aims and significance of the project. Where relevant, state the specific hypothesis to be tested. Also provide a brief description of current research/literature review, a justification as to why this research should proceed and an explanation of any expected benefits to the community.* [No more than 500 words]

Although having diagnostic categories for depression is useful in a clinical setting, community surveys suggest that depressive disorders exist on a fluid continuum, rather than as discrete categories. Depressive symptoms that fall short of meeting diagnostic criteria (variously termed sub-threshold, sub-clinical, sub-syndromal, mild, or minor depression) are common, cause significant functional disability, increase the risk of

developing major depressive disorder, and have considerable economic costs. For a particular individual, the disability from sub-threshold depression is lower than for a depressive disorder; however, the burden of disability for the population as a whole is substantial for sub-threshold depression because of its greater prevalence. Given that depressive disorders were the leading cause of disability burden globally in 2001, depressive symptoms falling short of a disorder are of major public health significance.

Although several trials have investigated treatments for sub-threshold depression, they have involved intervention by health professionals. An approach that does not require clinical resources is preferable. This is because there is already a large group of people with major depression who do not receive treatment, and treating these people deserves priority over those with sub-threshold symptoms. An alternative approach is self-help that can be applied by individuals affected without the need for professional guidance. Self-help approaches for depression are commonly used, particularly for milder forms of depression and are perceived as helpful by the public. However, some self-help methods in common use may not be helpful and are probably harmful (e.g. substance use).

Promotion of effective self-help strategies could be used as a cost-effective way of reducing sub-threshold depressive symptoms. Health promotion campaigns on other major sources of disease burden, such as heart disease and cancer, routinely include information on actions individuals can take to reduce risk (e.g. do not smoke, avoid salt and exercise more). This approach could be applied to individuals with elevated levels of depressive symptoms, with the aim of reducing symptoms and the risk of progressing to a depressive disorder. Intervening early could also reduce functional impairment and prevent progression to other undesirable outcomes such as harmful use of substances.

The proposed project takes an innovative approach by not placing demands on already-stretched clinical services. It adopts a health promotion approach already widely utilised in the physical health field, and applies it to depression, an Australian National Health Priority Area. This approach has the potential to reduce the distribution of depressive symptoms across the whole Australian community, resulting in significant reductions in population disability burden and fewer individuals experiencing a depressive disorder.

1.3 METHOD *Provide an outline of the proposed method, including details of the recruitment strategy and data collection techniques, the tasks participants will be asked to do, the estimated time commitment involved, and how data will be analysed*. [No more than 500 words]

The project will be a randomised controlled trial of internet users with depressive symptoms who do not meet diagnostic criteria for a depressive disorder (sub-threshold depression) and who are not already receiving treatment. The study will follow the requirements of the CONSORT statement for randomized trials and the Checklist for Reporting Results of Internet E-Surveys.

Participants will be recruited to the project website (http://www.moodmemos.com) by advertising on Google, links from depression and mental health websites and blogs, and posts on mental health forums. See Appendix A for a letter proforma asking whether webmasters would like to put a link to the moodmemos.com website on their website. An online screening questionnaire (Patient Health Questionnaire-PHQ9; Kroenke & Spitzer, 2002) will assess level of depressive symptoms and probable diagnosis of major depressive disorder. Participants who have sub-threshold depression symptoms will be provided with a plain language statement and invited to participate. They will be asked to complete a number of online questionnaires assessing psychological distress (K10; Kessler et al., 2002), functioning (Work and Social Adjustment Scale; Mundt et al., 2002) unpleasant experiences that may have caused depression (List of Threatening Experiences; Brugha et al., 1985), coping strategies, knowledge about depression, and sociodemographics (see Appendix B for all questionnaires), and to provide a name and email address. Those who meet admission criteria will be randomised to receive the active intervention or a control of educational information about depression. The intervention will be run on the internet and will be automated as much as possible through php computer scripts. The active group will receive email messages based on strategies thought to be effective and feasible by depression experts (Morgan & Jorm, 2009). See Appendix C for the text of each active message and an example of the layout of the email.

The control group will receive email messages based on information about depressive disorders, such as prevalence and risk factors. See Appendix D for the text of each control message and an example of the layout. The intervention will run for 6 weeks with emails sent twice a week. In addition to baseline, assessments will take place midway through the intervention, at the end of the intervention and 6 months after the end of the intervention. Total time commitment is estimated to be no more than 3 hours, including time to read each email as well as time taken to complete the four assessments.

Continuous outcomes (e.g. depressive symptoms) will be evaluated using mixed effects regression models, and dichotomous outcomes (e.g. presence of a depressive disorder) will be evaluated using logistic regression expressed as odds ratios with their 95% confidence intervals.

Brugha, T., P. Bebbington, et al. (1985). "The List of Threatening Experiences: a subset of 12 life event categories with considerable long-term contextual threat." Psychological Medicine 15(1): 189-94.

Kessler, R. C., G. Andrews, et al. (2002). "Short screening scales to monitor population prevalences and trends in non-specific psychological distress." Psychological Medicine 32(06): 959-976

Kroenke, K. and R. L. Spitzer (2002). "The PHQ-9: A new depression diagnostic and severity measure." Psychiatric Annals 32(9): 509-515.

Morgan, A. J. and A. F. Jorm (2009). "Self-help strategies that are helpful for sub-threshold depression: A Delphi consensus study." Journal of Affective Disorders 115: 196-200.

Mundt, J. C., I. M. Marks, et al. (2002). "The Work and Social Adjustment Scale: a simple measure of impairment in functioning." The British Journal of Psychiatry 180(5): 461-464.

1.4 USE OF INDEPENDENT CONTRACTORS *Will parts of this project be carried out by independent contractors? (e.g. interviewing, questionnaire design and analysis, sample testing, etc)*

☐ YES ☒ NO

If YES, confirm that the independent contractor will be engaged on the basis of relevant qualifications and experience and will receive from the Responsible Researcher, a copy of the approved ethics protocol and be made aware of their responsibilities arising from it. [The responsibility for effective oversight and proper conduct of the project remains with the Responsible Researcher]

1.5 MONITORING

(a) *How will researchers monitor the conduct of the project to ensure that it complies with the protocols set out in this application, the University's human ethics guidelines and the National Statement on Ethical Conduct in Human Research?* [Address, in particular, cases where several people are involved in recruiting, interviewing or administering procedures, or when the research is being carried out at some distance from the Principal Researcher (i.e. interstate or overseas)]

All the people carrying out the research are familiar with the National Statement.

(b) *For student research projects how will the student be supervised to ensure they comply with the protocols? If the student is working overseas, provide additional details of any local supervision arrangements.*

Regular supervision meetings will be held with the student approximately every three weeks, and the supervisors are both available for advice when needed outside these meeting times.

2. PARTICIPANT DETAILS

2.1 DOES THE RESEARCH *SPECIFICALLY TARGET*: [Tick as many as applicable]

		YES	NO
a.	students or staff of this University	☐	☒
b.	adults (over the age of 18 years and competent to give consent)	☒	☐
c.	children/legal minors (anyone under the age of 18 years)	☐	☒
d.	the elderly	☐	☒
e.	people from non-English speaking backgrounds	☐	☒
f.	pensioners or welfare recipients	☐	☒
g.	anyone intellectually or mentally impaired who cannot provide consent	☐	☒
h.	anyone who has a physical disability	☐	☒
i.	patients or clients of professionals	☐	☒
j.	anyone who is a prisoner or parolee	☐	☒
k.	a ward of the state	☐	☒
l.	any other person whose capacity to give informed consent may be compromised	☐	☒
m.	Aboriginal and/or Torres Strait Islander people and/or communities	☐	☒
n.	other collectives where a leader or council of elders may need to give consent	☐	☒

2.2 NUMBER, AGE RANGE AND SOURCE OF PARTICIPANTS
Provide number, age range and source of participants.

We will aim for a minimum of 1200 participants aged 18 or over from the following countries: Australia, New Zealand, UK, Ireland, Canada and USA. These countries were chosen because the research investigating appropriate self-help strategies was limited to these English-speaking countries.

2.3 JUSTIFICATION OF PARTICIPANT NUMBERS [The quality and validity of research is an essential condition of its ethical acceptability (refer National Statement)]. *Where applicable, provide a justification of sample size (including details of statistical power of the sample, where appropriate), explaining how this sample size will allow the aims of the study to be achieved.*

According to Cohen (1992), a sample of 393 per condition gives 80% power to detect a small effect size (0.2 standard deviations between conditions). Because depression Internet trials have a median dropout rate of 33% (Christensen et al., 2009), a total sample of 1200 participants will be aimed for.

Cohen, J. (1992). A power primer. Psychological Bulletin 112: 155-159.
Christensen H, Griffiths KM, Farrer L. Adherence in internet interventions for anxiety and depression. J Med Internet Res. 2009;11(2):e13.

2.4 PARTICIPANT RECRUITMENT

(a) *Please indicate the method of recruitment by ticking the appropriate boxes. Tick all that apply.*

**Mail out -** see below	☐	**Email -** see below	☐	**Telephone**	☐
**Advertisement -** see below	☒	**Recruitment carried out by third party** (eg. employer, doctor) – see below	☐	**Recruitment carried out by researcher/s**	☐
**Contact details obtained from public documents (**eg. phone book)	☐	**Contact details obtained from private sources** (eg. employee list, membership database) – see below	☐	**Personal contacts**	☐
**Participants from a previous study**	☐	**Snowball** (participants suggest other potential participants)	☐	**Other** *(Please explain in no more than 50 words)*:	☐

- *If using a* ***mail out*** *or* ***email*** *who will be distributing it?*
  N/A

- *If using an* ***advertisement****:*
  - *explain where will it be placed?*[e.g. on waiting room wall, in newspaper, in newsletter]
    Advertisements will be placed in Google and Yahoo attached to search terms such as 'depression'. Links to the project website will also be placed in mental health forums and mental health websites, with the permission of forum administrators and webmasters.
  - *have you attached a copy?* Please see Appendix E for the Google/Yahoo advertisements.

  Yes ☒ No ☐ NA ☐ *If "No" please explain (no more than 50 words):*

- *If recruitment is to be conducted by a* ***third party****, (eg employer, doctor) have you attached an approval letter?*

  - requesting their assistance?[yes, no or not applicable]
  Yes ☐ No ☐ NA ☐ *If "No" please explain (no more than 50 words):*

  - confirming their willingness to assist?
  Yes ☐ No ☐ NA ☐ *If "No" please explain (no more than 50 words):*

  - that has been drafted for the third party to send to potential participants?
  Yes ☐ No ☐ NA ☐ *If "No" please explain (no more than 50 words):*

- *If contact details are to be obtained from* ***private sources****, have you attached an approval letter?*
  Yes ☐ No ☐ *If "No" please explain (no more than 50 words):*

(b) *Describe how, by whom, where potential participants are to be identified or selected for this research.*

Potential participants will be anonymous internet users who voluntarily complete an online depression-screening questionnaire and have results indicating sub-threshold depression.

(c) *Describe how, by whom, where potential participants are to be approached or invited to take part in this research.*

Participants will be internet users who have searched for information or help about depression symptoms through Google or Yahoo, or mental health websites, blogs, or forums and have clicked through to the moodmemos.com website.

2.5 DEPENDENT RELATIONSHIPS

[The issue of research involving persons in dependent or unequal relationships (e.g. teacher/student, doctor/patient, student/lecturer, client/counsellor, warder/prisoner, and employer/employee) is discussed in Sections 2 and 4.3 of the National Statement. Such a relationship may compromise a participant's ability to give consent which is free from any form of pressure (real or implied)]. *Are any of the participants in a dependent relationship with any of the researchers, particularly those involved in recruiting for or conducting the project?*

☐ YES ☒ NO *(If YES, explain the dependent relationship and the steps to be taken by the researchers to ensure that participation is purely voluntary and not*

influenced by the relationship in any way.

2.6 PAYMENT OR INCENTIVES OFFERED TO PARTICIPANTS
Do you propose to pay, reimburse or reward participants?

☐ YES ☒ NO *(If YES, how, how much and for what purpose? Please justify the approach)*

2.7 DECEPTION OR CONCEALMENT
[Limited disclosure, deception and active concealment are discussed in Section 2.3 of the National Statement. Essentially the practice is not considered ethical unless there are compelling reasons given for its use] *Will the true purpose of the research, or the collection of data itself, be concealed from participants or will participants in any way be deceived?*

☐ YES ☒ NO

If you answered YES, provide a clear justification. [You will also need to provide participants with details of the deception in a debriefing (refer 3.4) and give them the opportunity to withdraw their data if they wish to do so.]

3. RISK AND RISK MANAGEMENT

3.1 STUDY PROFILE –DOES THE RESEARCH INVOLVE THE FOLLOWING:
[Tick as many as apply. Provide details in methodology –section 1.5 and attach information where indicated]

	YES	NO
• use of questionnaires designed by the researcher ***(attach a copy)***	☒	☐
• use of standard survey instruments ***(attach a copy)***	☒	☐
• use of on-line surveys ***(attach printout of screen information)****	☒	☐
• use of interviews ***(attach the list of interview questions)***	☐	☒
• use of focus groups ***(attach the list of focus group topics/questions)***	☐	☒
• observation of participants without their knowledge	☐	☒
• covert observation	☐	☒
• audio-taping interviewees or events	☐	☒
• video-taping interviewees or events	☐	☒
• access to personal and/or confidential data (including student, patient or client data) without the participant's specific consent	☐	☒
• administration of any stimuli, tasks, investigations or procedures which may be experienced by participants as physically or mentally painful, stressful or unpleasant during or after the research process	☐	☒
• performance of any acts which might diminish the self-esteem of participants or cause them to experience embarrassment, regret or depression	☐	☒
• research about participants involved in illegal activities	☐	☒
• research conducted in an overseas setting	☒	☐
• administration of any substance or agent	☐	☒
• use of non-treatment or placebo control conditions	☒	☐
• collection of body tissues or fluid samples	☐	☒
• collection and/or testing of DNA samples	☐	☒

* The surveys have not yet been put online, however they will only involve the questionnaires already provided

See Appendix B for the questionnaires.

3.2 POTENTIAL RISKS TO PARTICIPANTS
Identify, as far as possible, all potential risks to participants (e.g. physical, psychological, social, legal or economic etc.), associated with the project and the setting (e.g. overseas) in which the project is conducted. It may be useful to consider the study profile above and your response to participant details in section 2

Minimal discomfort is anticipated for participants. The intervention will not be an imposition on participants, as they will be seeking assistance for their symptoms. There is a small chance that completing the questionnaires may cause distress, however, a systematic review of the evidence on whether participation in psychiatric research causes distress concluded that a minority of participants become distressed, but there is no evidence of long-term harm. Positive reactions are more common than negative ones (Jorm et al., 2007). Furthermore, questionnaires that screen for depression are already commonly used on the internet, for example on beyondblue's website.

There is also a risk that some participants will experience worse mental health despite the intervention (see 3.6 for management of this risk). There is a risk that some participants may attempt suicide, although we believe this is a small risk as research indicates that those with sub-threshold depression are less likely to report suicidal symptoms than those with major depression (Karlsson et al., 2007).

Jorm, A.F., Kelly, C.M. & Morgan, A.J. (2007). Participant distress in psychiatric research: a systematic review. *Psychological Medicine*. 37(7):917-26.

Karlsson, L., et al., Differences in the clinical characteristics of adolescent depressive disorders. Depression and Anxiety, 2007. 24(6): p. 421-432.

3.3 MANAGING POTENTIAL RISKS

Describe what measures you have in place to minimize these potential risks to participants and to ensure that support is available if needed. [Depending on risks, participants may need additional support (e.g. external counseling) during or after the study]

Some potential participants may screen positive for major depression. The screening questionnaire is a brief self-report measure that cannot give a diagnosis on its own, but can indicate whether a diagnosis is probable. In this instance, participation in the project may not be the most appropriate strategy to improving mental health. These participants will not be eligible for participation; rather, they will be informed that they may have major depression and should seek appropriate professional help. Appropriate sources of help will be provided on the website (see Appendix F).

3.4 DEBRIEFING (if applicable)

What debriefing will participants receive following the study and when? (Attach a copy of any written material or statement to be used in such a debriefing, if applicable). [Participants may need to talk about the experience of being involved in the study with the researchers, as well as learn more about the aims of the research]

Not applicable.

3.5 BENEFITS COMPARED TO POTENTIAL RISKS

Outline the benefits of the study to the community (and participants, if applicable), relative to the potential risks to participants

As participation is limited to those who are not being treated for their depression, participation may benefit the participants through improving their depression symptoms and mental wellbeing. The study may also benefit the community through adding to knowledge about ways to reduce or prevent depression, knowledge which can be promoted to the community as a whole. As the intervention involves benign strategies thought to be helpful by experts, and participants can withdraw if their symptoms worsen, the benefits outweigh the potential risks. Even if the results are non-significant, the study is adequately powered to be able to rule out the potential effectiveness of this approach. This will benefit the community by protecting them against the mounting of public health campaigns with no evidence base.

3.6 MANAGING ADVERSE / UNEXPECTED OUTCOMES

Describe what measures you have in place in the event that participants experience adverse effects arising from their involvement in the project (e.g. adverse drug reaction, revelation of illegal activity, or unexpected distress due to questioning)

Despite the intervention, some participants may develop major depression or become acutely suicidal. To manage this risk, participants will be encouraged throughout the project to seek help from a professional if they feel they are getting worse. To facilitate this, a section of the website will provide information on seeking professional help for each country involved. Participants will also receive feedback from their survey results on their level of symptoms, and encouraged to seek professional help if their symptom levels indicate major depression. Currently many people with depression do not seek and/or receive help for their symptoms. The links provided on the website will channel potential participants using the Internet for mental health information to appropriate sources of help. Participants will be informed they can discontinue participation at any time.

3.7 POTENTIAL RISKS TO RESEARCHERS

Will there be any significant risks to researchers associated with the project and the setting (e.g. overseas) in which the project is conducted. (e.g. personal safety, health, emotional well being)? [Refer to the University's Environmental Health & Safety Manual for more information]

☐ YES ☒ NO *(If YES, how will such risks be addressed)*

4. INFORMATION FOR PARTICIPANTS AND INFORMED CONSENT

Before research is undertaken, the informed and voluntary consent of participants (and other properly interested parties) is generally required (refer Section 2 of the National Statement for more details). Information needs to be provided to participants at their level of comprehension about the purpose, methods, demands, risks, inconveniences, discomforts and possible outcomes of the research. Such information is often provided in a written **Plain Language Statement**. Each

participant's consent needs to be clearly established (e.g. by using a signed **Consent Form**, returning an anonymous survey or recording an agreement for interview).

4.1 PROVIDING INFORMATION FOR PARTICIPANTS

(a) *Will you be providing participants with information in a written Plain Language Statement?*

☒ YES ☐ NO *(If NO, provide details of the protocol you will use to explain the research project to participants and invite their participation?)*

(b) *Will arrangements be made to ensure that participants who have difficulty understanding English can comprehend the information provided about the research project?*

☐ YES ☒ NO *(If YES, what arrangements have been made? If NO, give reasons.* Only participants who have sufficient English skills will be eligible to participate.

4.2 PLAIN LANGUAGE STATEMENT (IF APPLICABLE)

CONFIRM THAT THE PLAIN LANGUAGE STATEMENT WILL:

		YES	NOT APPLICABLE
1.	be printed on University of Melbourne letterhead	☒	
2.	include clear identification of the University, the Department(s) involved, the project title, the Principal and Other Researchers (including contact details), and the study level if it is a student research project.	☒	
3.	provide details of the purpose of the research project	☒	
4.	provide details of what involvement in the project will require (e.g., involvement in interviews, completion of questionnaire, audio/video-taping of events), and estimated time commitment	☒	
5.	provide details of any risks involved and the procedures in place to minimise these.	☒	☐
6.	advise that the project has received clearance by the HREC	☒	
7.	(if the sample size is small), confirm that this may have implications for protecting the identity of the participants	☐	☒
8.	include a clear statement that if participants are in a dependent relationship with any of the researchers that involvement in the project will not affect ongoing assessment/grades/management or treatment of health (if relevant)	☐	☒
9.	state that involvement in the project is voluntary and that participants are free to withdraw consent at any time, and to withdraw any unprocessed data previously supplied	☒	
10.	provide advice as to arrangements to be made to protect confidentiality of data, including that confidentiality of information provided is subject to legal limitations (see ** below)	☒	
11.	provide advice as to whether or not data is to be destroyed after a minimum period (if relevant)	☒	☐
12.	provide in the footer, the project HREC number, date and version of the PLS	☒	
13.	provide advice that if participants have any concerns about the conduct of this research project that they can contact the Executive Officer, Human Research Ethics, The University of Melbourne, ph: 8344 2073; fax 9347 6739	☒	

[**Re 10 – it is possible for data to be subject to subpoena, freedom of information request or mandated reporting by some professions. Depending on the research proposal you may need to specifically state these limitations]

PLEASE ATTACH A COPY OF THE PLAIN LANGUAGE STATEMENT TO YOUR APPLICATION

Please see Appendix G for the Plain Language Statement.

4.3 OBTAINING CONSENT

(a) *How will each participant's consent be established?*

**By signing and returning a Consent Form –** *see 4.4 below*	☐	**By returning an anonymous survey**	☐
**Via a verbal agreement**	☐	**Via a person with lawful authority to consent** (eg. parent, doctor) – *see 4.3(b) below*	☐
**Via a recorded agreement for interview**	☐	**Other** *(Please describe in no more than 50 words)*: Participants will select a tick box online indicating their informed consent to participate. This consent will be stored in the data file. "I have read the information sheet for this study and agree to participate under the conditions described. YES/NO"	☒

(b) *If participants are unable to give informed consent, explain who will consent on their behalf and how such consent will be obtained.*

If participants are unable to give informed consent they will not be eligible to participate.

4.4 CONSENT FORM (IF APPLICABLE)

(N/A)

CONFIRM THAT THE CONSENT FORM WILL:

		YES	NOT APPLICABLE
1.	be printed on University of Melbourne letterhead	☐	
2.	include the title of the project and names of researchers	☐	
3.	state that the project is for research purposes	☐	
4.	state that involvement in the project is voluntary and that participants are free to withdraw at any time, and free to withdraw any unprocessed identifiable data previously supplied	☐	
5.	outline particular requirements of participants including, for example, whether interviews are to be audio and/or video-taped	☐	☐
6.	include arrangements to protect the confidentiality of data	☐	
7.	include advice that there are legal limitations to data confidentiality (see below)**	☐	
8.	(if the sample size is small) confirm that this may have implications for protecting the identity of the participants	☐	☐
9.	(once signed and returned) be retained by the researcher	☐	

[**Re 7 – it is possible for data to be subject to subpoena, freedom of information request or mandated reporting by some professions. Depending on the research proposal you may need to specifically state and explain these limitations]

PLEASE ATTACH A COPY OF THE CONSENT FORM TO YOUR APPLICATION

5. PRIVACY AND CONFIDENTIALITY

Privacy can be described as "…a complex concept that stems from a core idea that individuals have a sphere of life from which they should be able to exclude any intrusion." A major application of the concept of privacy is information privacy: the interest of a person in controlling access to and use of any information personal to that person. 'Confidentiality', a narrower more specific term than 'privacy' refers to the legal and ethical obligation that arises from a relationship in which a person receives information from or about another.

At the Commonwealth level, the collection, storage, use and disclosure of personal information by Commonwealth agencies is regulated by the *Privacy Act 1988*. Sections 95 and 95A of the Act are of particular relevance to researchers. There is regulation at State and Territory level in the form of legislation related to privacy generally or the administration of agencies, or administrative codes of practice. In Victoria, the *Health Records Act 2001* regulates health information handled by the Victorian public sector and private sector, while the Information Privacy Act 2000 regulates the collection and handling of non-health-related personal information. The National Statement states that an HREC must be satisfied that a research proposal conforms to all relevant Commonwealth, State or Territory privacy legislation or codes of practice]

5.1 ACCESSING PERSONAL INFORMATION

[Personal Information' includes names, addresses, or information/opinion about an individual whose identity is apparent, or can reasonably be ascertained, from the information/opinion. It also includes Health Information (e.g. health opinions, organ donation or genetic information) and Sensitive Information (e.g. political views, sexual preferences, criminal records)]

*Is there a requirement for the researchers to obtain **Personal Information** (either identifiable or potentially*

*identifiable) about individuals **without their consent**?*

		YES	NO
a)	from Commonwealth departments or agencies?	☐	☒
b)	from State departments or agencies?	☐	☒
c)	from Other Third Parties, such as non-government organisations?	☐	☒

If you answered YES to (a), (b) or (c), you will need to complete Module P and attach it to this application

5.2 REPORTING PROJECT OUTCOMES

(a) *Will the project outcomes be made public at the end of the project?*

☒ YES ☐ NO

(If YES, give details of how the results will be made public (eg in journal articles book, conference paper, the media, working paper or other). If NO, explain why not.

The findings will be disseminated to researchers and health professionals in journals and at conferences. An effort will be made to disseminate the findings to the public through the media.

(b) *Will a report of the project outcomes be made available to participants at the end of the project?*

☒ YES ☐ NO

(If Yes, give details of the type of report and how it will be made available. If No, explain why not.

A report of the project outcomes will be made available on the website.

5.3 WILL THE RESEARCH INVOLVE:

	YES	NO
• complete anonymity of participants (i.e., researchers will not know the identity of participants as participants are part of a random sample and are required to return responses with no form of personal identification)?	☐	☒
• de-identified samples or data (i.e., an irreversible process whereby identifiers are removed from data and replaced by a code, with no record retained of how the code relates to the identifiers. It is then impossible to identify the individual to whom the sample of information relates)?	☐	☒
• potentially identifiable samples or data (i.e., a reversible process in which the identifiers are removed and replaced by a code. Those handling the data subsequently do so using the code. If necessary, it is possible to link the code to the original identifiers and identify the individual to whom the sample or information relates)?	☒	☐
• participants having the option of being identified in any publication arising from the research?	☐	☒
• participants being referred to by pseudonym in any publication arising from the research?	☐	☒

• any other method of protecting the privacy of participants? *Please describe:*

Note that where the sample size is very small, it may be impossible to guarantee anonymity/confidentiality of participant identity. Participants involved in such projects need to be clearly advised of this limitation in the Plain Language Statement.

6. DATA STORAGE, SECURITY AND DISPOSAL

6.1 DATA STORAGE

Does data storage comply with the University policy? [University of Melbourne Policy on the Management of Research Data and Records is available at: **http://www.unimelb.edu.au/records/research.html** The Joint NHMRC/AVCC Statement and Guidelines on Research Practice is available at: **http://www.nhmrc.gov.au/funding/policy/researchprac.htm**]

☒ YES ☐ NO *(If NO, please explain.)*

6.2 DATA SECURITY

(a) *Will the Principal Researcher be responsible for security of data collected?*

☒ YES ☐ NO *(If NO, please provide further details. You may also use this space to explain any differences between arrangements in the field, and on return to campus.)*

(b) *Will data be kept in locked facilities in the Department through which the project is being conducted?*

☒ YES ☐ NO *(If NO, please explain how and where data will be held, including any arrangements for data security during fieldwork.)*

(c) *Which of the following methods will be used to ensure confidentiality of data?*
(select all options that are relevant)

- data and codes and all identifying information to be kept in separate locked filing cabinets ☒
- access to computer files to be available by password only ☒
- access by named researcher(s) only ☒
- other *(please describe)* ☐

(d) *Will others besides the researchers associated with this project have access to the raw data?*

☐ YES ☒ NO *(If YES, please explain who and for what purpose? What is their connection to the project?)*

6.3 DATA RETENTION AND DISPOSAL

[Research data and records should be maintained for as long as they are of *continuing value* to the researcher and as long as recordkeeping requirements such as patent requirements, legislative and other regulatory requirements exist. The minimum retention period for research data and records is five years after publication, or public release, of the work of the research as stated in the University of Melbourne *Code of Conduct for Research*. If the project involves clinical trial(s), the data should be kept for a minimum of 15 years (refer to Section 3.3 of the National Statement for further details)]

Specify how long materials (e.g. files, audiotapes, questionnaires, videotapes, photographs) collected during the study will be retained after the study and how they will ultimately be disposed of.

A data file will be kept for at least 5 years after publication or as long as interest in the data continues.

7. POTENTIAL CONFLICT OF INTEREST

7.1 POTENTIAL CONFLICT OF INTEREST

Is there any affiliation or financial interest for researchers in this research or its outcomes or any circumstances which might represent a perceived, potential or actual conflict of interest?

☐ YES ☒ NO *(If YES, give brief details?)*

[If you have declared a potential conflict of interest, you should include an appropriate comment on the Plain Language Statement and Consent Form]

7.2 COMPLIANCE WITH THE CODE OF CONDUCT FOR RESEARCH

[University researchers must disclose and manage Conflict of Interest in accord with the provisions of the University's *Code of Conduct for Research*. See http://www.unimelb.edu.au/ExecServ/Statutes/r171r8.html]

Is the Conflict of Interest noted above in section 7.1 being managed in accordance with the Code of Conduct?

☐ YES ☐ NO ☒ Not Applicable

8. DECLARATION BY RESEARCHERS

The information contained herein is, to the best of our knowledge and belief, accurate.

We have read the University's current human ethics guidelines, and accept responsibility for the conduct of the procedures set out in the attached application in accordance with the guidelines, the University's Code of Conduct for Research and any other condition laid down by the University of Melbourne's Human Research Ethics Committee or its Sub-Committees. We have attempted to identify all risks related to the research that may arise in conducting this research and acknowledge our obligations and the rights of the participants. We have the appropriate qualifications, experience and facilities to conduct the research set out in the attached application and to deal with any emergencies and contingencies related to the research that may arise.

If approval is granted, the project will be undertaken in strict accordance with the approved protocol and relevant laws, regulations and guidelines.

We, the researcher(s) agree:
- *To only start this research project after obtaining final approval from the Human Research Ethics Committee (HREC);*
- *To only carry out this research project where adequate funding is available to enable the project to be carried out according to good research practice and in an ethical manner;*
- *To provide additional information as requested by the HREC;*
- *To provide progress reports to the HREC as requested, including annual and final reports;*
- *To maintain the confidentiality of all data collected from or about project participants, and maintain security procedures for the protection of privacy;*
- *To notify the HREC in writing immediately if any change to the project is proposed and await approval before proceeding with the proposed change;*
- *To notify the HREC in writing immediately if any adverse event occurs after the approval of the HREC has been obtained;*
- *To agree to an audit if requested by the HREC;*
- *To only use data and any tissue samples collected for the study for which approval has been given;*

We have read the National Statement on Ethical Conduct in Human Research and agree to comply with its provisions.

All researchers associated with this project must sign

Researchers' Name (please PRINT)	Signature	Date
PROF TONY JORM	Anthony Jorm	5/10/09
PROF ANDREW MACKINNON	A. J. Mackinnon	5/10/09
MS AMY MORGAN	Amy Morgan	5/10/09

9. DECLARATION BY DEPARTMENTAL HUMAN ETHICS ADVISORY GROUP (HEAG)

DATE APPLICATION RECEIVED: / / *HEAG NO:*

☐ *TECHNICAL REVIEW COMPLETED* ☐ *ETHICAL REVIEW COMPLETED*

The HEAG has reviewed this project and considers the methodological/technical and ethical aspects of the proposal to be appropriate to the tasks proposed and recommends approval of the project. The HEAG considers that the researcher(s) has/have the necessary qualifications, experience and facilities to conduct the research set out in the attached application, and to deal with any emergencies and contingencies that may arise. [Note: If the HEAG Chair is also a principal researcher for this project, the declaration should be signed by another authorised member of the HEAG]

Comments/Provisos:

Name of HEAG Chair (in BLOCK LETTERS)	
Signature	
Date	

10. DECLARATION BY HEAD OF DEPARTMENT

DATE APPLICATION RECEIVED: / / *HEAG NO:*

☐ *TECHNICAL REVIEW COMPLETED* ☐ *ETHICAL REVIEW COMPLETED*

I have reviewed this project and consider the methodological, technical and ethical aspects of the proposal to be appropriate to the tasks proposed and recommend approval of the project. I consider that the researcher(s) has/have the necessary qualifications, experience and facilities to conduct the research set out in the attached application, and to deal with any emergencies and contingencies that may arise. [Note: If the Head of Department is also a principal researcher for this project, the declaration should be signed by another authorised member of the Department]

This project has the approval and support of this Department/School/Centre.

Name of Head (in BLOCK LETTERS)	
Signature	
Date	

11. WHEN COMPLETE

When this form has been completed and finalised it should be attached to the coversheet section of the application completed in Themis Research and then submitted to the nominated Human Ethics Advisory Group for review.

THE UNIVERSITY OF MELBOURNE
HUMAN RESEARCH ETHICS COMMITTEE

REQUEST FOR AMENDMENT

1. ADMINISTRATION DETAILS

**DATE OF SUBMISSION:**	**02-Jun-2011**	**ETHICS ID:**	**0931313.3**
**APPLICATION TYPE:**	**Project Application**		
**RESPONSIBLE HEAG:**	Centre for Youth Mental Health	**HESC:**	Behavioural and Social Sciences
**ADMINISTERING DEPARTMENT:**	Psychiatry	**ADMINISTERING CENTRE:** (if applicable)	Orygen Youth Health Research Centre

2. PROJECT DETAILS

Title: Promotion of self-help strategies for sub-threshold depression: An e-mental health RCT

Brief Description: Depressive symptoms are common, impair individual functioning, and may develop into major depression. Self-help strategies, such as exercise or taking time off work, are well-liked and often used by those with depressive symptoms. However, some strategies are not as effective as others, and some may be harmful (e.g. substance use). Previous research has identified a set of helpful and feasible self-help strategies. This project will test the feasibility of promoting these strategies via email messages to individuals with a high level of depressive symptoms. The aim is to reduce the overall level of depressive symptoms as well as the number of individuals developing major depression.

Project Approval Period: **From**: 04-Jun-2009 **To**: 31-Dec-2011

3. PERSON DETAILS

Role	Name	Person Type	Department/ Organisation	Phone/ Alternate Phone	Email/ Alternate Email
Co researcher	Prof Andrew Mackinnon	Staff	554 - Psychiatry		Andrew.Mackinnon@unimelb.edu.au
Responsible Researcher	Prof Anthony Jorm	Staff	554 - Psychiatry	93423747	ajorm@unimelb.edu.au
Student Researcher	Amy Morgan	Student	554 - Psychiatry	0402880802Alt Phone:9342 3764	a.morgan@pgrad.unimelb.edu.au

4. REQUEST FOR AMENDMENT DETAILS

4.1 Nature of and reason for amendment

We propose three amendments to the project.

1) We wish to amend the inclusion criteria of the project, to allow participation from anyone interested in the study regardless of their depression status (phase two). Previously, participation was restricted to adults with sub-threshold symptoms of depression, who were not receiving depression treatment (phase one). We would like to open up the study to those with full threshold depression, as well as those with any number of symptoms. Undergoing concurrent treatment for depression will also not be an exclusion criterion, because the intervention does not interfere with psychological or medical treatment. This recognizes that participants will be more likely to be in treatment if they have full-threshold depression.

We wish to make this amendment because preliminary analyses of the study show that the Mood Memo emails change self-help coping behaviour, and depressive symptoms to a smaller extent. We hypothesise that the effect on depressive symptoms will be greater for those who are more depressed at the beginning of the study than for those with sub-threshold depression. We think this is probable because effect sizes are typically greater for treatment studies than for prevention studies (Jané-Llopis, Hosman, et al., 2003). Furthermore, there was significant interest in participating from individuals who were already in treatment or who had 'full' depression, and it would be worthwhile investigating whether the Mood Memos had any effects additional to treatment effects.

We plan to analyse the data collected with the changed inclusion/exclusion critieria separately from that already collected in phase one. This is feasible because of the number of participants recruited to date. This will also preclude any suggestion that the trial is being extended to ‘chase’ statistical significance.

We will continue to advertise the study and recruit participants in the same manner as before (primarily through Google advertising). We will aim for a sample size of 1600 participants in this second phase of the study. This is based on a power analysis indicating that a sample of 393 per condition would give 80% power to detect a small effect size (0.2 standard deviations between conditions) on a continuous outcome measure assuming a correlation of 0.5 between pre- and post-test scores. As approximately 50% of participants in phase one did not complete the post-test assessment, a total sample of 1600 allows for a similar rate of attrition. 1600 participants is a feasible target given that 1336 participants with sub-threshold depression were recruited in phase one.

To clarify, the inclusion criteria will now be:
a. 18 years or older
b. Access to the internet at least once per week
c. Resident of Australia, New Zealand, Canada, Ireland, United Kingdom, USA
d. Any score on the PHQ-9 depression questionnaire
e. Informed consent

2) The second change we propose is to send an additional reminder email requesting the participant to complete post- and follow-up assessments, bringing the number of reminders to two. This is because up to 50% of participants did not complete assessments, and the reminder email resulted in about 25% more responses. Therefore, a second reminder may lead to a significantly higher response rate.

3) The third change we propose is to amend the wording of the help-seeking question asked at mid-point, post intervention and follow-up. This is so that we can collect more specific information about treatments received by participants. The question will be changed from:
"Have you visited a health professional (e.g. doctor, counsellor, psychologist) to help deal with your depression during the past [3 weeks] / [6 weeks] / [6 months]? (Yes/No)"
to:
"Which treatments for depression have you received from a professional during the past [3 weeks] / [6 weeks] / [6 months]? (multiple responses allowed)
• Nothing, just the consultation
• Psychological therapy
o Acceptance and commitment therapy
o Behaviour therapy
o Cognitive behaviour therapy
o Dialectical behaviour therapy
o Family therapy
o Mindfulness-based cognitive therapy
o Problem solving therapy
o Psychoanalytic psychotherapy
o Supportive psychotherapy or supportive counselling
o Not sure what type
• Medication
o Antidepressant medication (e.g. Zoloft, Lexapro)
o Antipsychotic medication (e.g. Zyprexa, Seroquel)
o Anti-anxiety medication (e.g. Valium, Xanax)

o Mood stabilising medication (e.g. Lithicarb)
o Stimulant medication (e.g. Ritalin, Modavigil)
o Not sure what type

Jané-Llopis, E., C. Hosman, et al. (2003). "Predictors of efficacy in depression prevention programmes: Meta-analysis." British Journal of Psychiatry 183(5): 384-397.

4.2 Impact on documentation

The plain language statement will be amended to reflect the change in inclusion criteria and will include a statement about the Mood Memos not being a substitute for professional help (see second paragraph in attachment).

4.3 Possible inconvenience or risks to participants

There are no changes to the content of the intervention hence no new risks are anticipated. However, there is a risk that participants may think the intervention is a substitute for professional treatment, which could be problematic for those with full depression. This could mean that participants may be less likely to seek professional help if they have not already done so, which could prolong their depressive episode.

4.4 Actions to be taken by researchers to reduce risks

The plain language statement will be amended to include a statement that the study is not a substitute for professional treatment or diagnosis and professional help is always recommended for depression. Furthermore, the following statement will be repeated in the footer of each Mood Memo email: "This email is not a substitute for professional treatment. Professional help is always recommended for depression."

Other open-access internet-based depression interventions (such as MoodGYM, http://www.moodgym.anu.edu.au) take a similar approach to managing the risk of depressed individuals using the program without professional supervision. MoodGYM is a well-regarded and widely used program, and it states the following on its website (http://www.moodgym.anu.edu.au/welcome/faq#doctor):

"The information provided throughout MoodGYM is intended for information and skill development purposes only. MoodGYM is not a substitute for seeking diagnosis and treatment from a qualified person. Always consult a medical practitioner or mental health professional if you require a diagnosis or treatment for depression, anxiety or other mental disorders. One reason this is important is that depression is sometimes caused by physical illness or certain medications and a doctor can check these out. Persons under age 16 with depressive symptoms should speak to their parents about seeking professional help for their depression."

4.5 Expected date of implementation of amendment to research

30-Jun-2011

4.6 Possible affect on funding arrangements

N/A

4.7 Possible implications for compliance with legislative requirements

N/A

5. ATTACHMENTS

Copies of the following need to be attached:

(a) copies of amended surveys, questionnaires or interview questions
(b) copies of the amended advertisement, plain language statement, and consent form
(c) any permission or approval letters required as a result of the proposed changes

6. SIGNATURES

RESPONSIBLE RESEARCHER

	Signature	Date
Prof A.F. Jorm		

HEAG/DEPARTMENT USE ONLY

Please tick ONE of the following

The HEAG/Head of Department recommends the amendment for approval by the HESC	
The HEAG approves this amendment as the project was previously approved as a minimal risk project or as a project-within program and the amendment presents no additional risks.	

Comments/Provisos:

	Signature	Date
Prof Henry Jackson*		

* If the HEAG Chair is also a researcher involved in this project, the Request for Amendment should be signed by another authorised member of the HEAG. If the Department does not have access to a HEAG the Request for Amendment should be signed by the Head of Department.
