# Supplementary material for: Self-Help for Depression via E-mail: A Randomised Controlled Trial of Effects on Depression and Self-Help Behaviour
Source: PLoS One. 2013 Jun 21;8(6):e66537. doi: 10.1371/journal.pone.0066537 (PMC3689826; doi:10.1371/journal.pone.0066537)
Supplement: Active emails S1 — Screenshots of the active group emails. (PDF) [file pone.0066537.s003.pdf]

Having trouble reading this email? View it in a web browser

mood memos

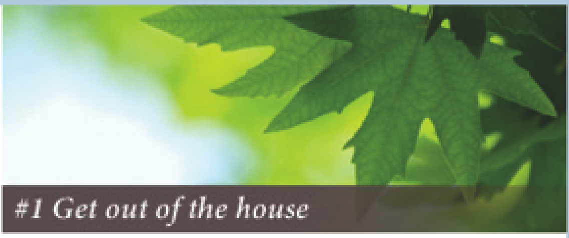

Dear Amy,

If you want to feel better, **make sure you get out of your home for at least a short time each day**.

Why?

- Depression can make you want to hide from the world and be on your own all day.
- But avoiding others will make you feel even more isolated and alone.
- Staying cooped up inside also strengthens your negative thinking patterns.
- Getting out of the house for at least a short time each day was recommended by more than 80% of therapists and people who have recovered from depression.

How?

- If you stay inside your home during the day, all evening, or on weekends, make a commitment to get outside for at least a short time.
- **Try to think of a reason to get out.** If you spread tasks (such as shopping or posting mail) over the week, you will always have a reason to leave the house.
- If you can go out with another person or a pet, that is even better.

But...

What if you can't think of a reason to leave? It doesn't matter what you do when you are out, for example, you could go for a walk around the block.

Commit

Commit to getting out of your home each day. Right now, set aside a minute to plan how you will achieve this goal for this week.

Imagine **when** and **where** you are going to get out. Write your goal down, or remember to tell someone your goal. You can also print this email as a reminder.

Example

“*This weekend I will get out of the house each day. I will visit the new shop in town on Saturday afternoon and on Sunday I will go for a brisk walk around the block after lunch.*”

© 2010 – 2011 MoodMemos.com | Orygen Youth Health | University of Melbourne | 35 Poplar Road Parkville VIC 3052 Australia

Amy you have received this email because you signed up to take part in the MoodMemos.com study. If you want to stop receiving emails from us and withdraw from the study, please unsubscribe here.

mood memos

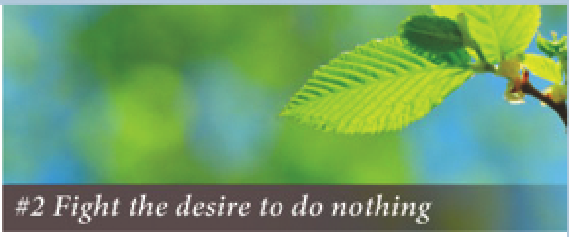

Dear Amy,

You can help yourself feel better by **keeping up with your daily tasks.**

Why?

- Depression makes it hard to get up and do things. However, avoiding daily tasks can make you feel worse because you feel guilty about it.
- Keeping active will prevent you from being preoccupied with negative thoughts and will help fight feelings of lethargy.
- Keeping up with your daily tasks was recommended by more than 80% of therapists and people who have recovered from depression.

How?

- Try to do some things you have let slide lately.
- For example, are the dishes piling up? Have you stopped brushing your teeth because it is too hard? Are your clothes in need of a good wash? Do you have bills that need paying? Have you been avoiding returning emails or phone calls?
- Try to tackle these things each day, for at least some part of the day.
- To help motivate yourself, give yourself a compliment or a reward when you have completed a task.

But...

You may be thinking that it is pointless to keep up with activities because you don't have any energy. Don't listen to those thoughts – just try to do as much as you can. Remember also not to be hard on yourself if there are times when you fall back into the habit of doing nothing.

Commit

Make a commitment to fight the desire to do nothing. Right now, set aside a minute to plan a goal you would like to achieve this week.

Imagine **when** and **where** you are going to do it. Write your goal down, or remember to tell someone your goal. You can also print this email as a reminder.

Example

“*This week I will do the dishes and clean up the kitchen every evening.*”

Past commitments

In the last Mood Memo we sent you, we encouraged you to commit to **getting out of your home each day**. *How did you go?*

© 2010 – 2011 MoodMemos.com | Orygen Youth Health | University of Melbourne | 35 Poplar Road Parkville VIC 3052 Australia

Amy you have received this email because you signed up to take part in the MoodMemos.com study. If you want to stop receiving emails from us and withdraw from the study, please unsubscribe here.

mood memos

#3 Set yourself a small goal and reward yourself for reaching it

Dear Amy,

If you are feeling a bit depressed, one way to feel better is to **set yourself a small goal and then reward yourself for reaching it.**

Why?

- People who are depressed often stop setting goals or set unachievable goals that are vague or unrealistic.
- Setting and achieving goals leads to a sense of completion and satisfaction.
- Setting goals is a way of fighting hopelessness and being positive about the future and was recommended by more than 80% of therapists and people who have recovered from depression.

How?

- Think about a small goal you would like to achieve.
- Have a clear purpose for why you want to achieve it.
- Make it specific and concrete so you know when it has been reached.
- Make it something that is possible for you to achieve.
- Give yourself a timeframe in which to achieve your goal (e.g. today, this week, this month).
- Think of a small reward you can give yourself when you reach your goal, as this can be extra motivation for reaching your goal.

But...

What if you can't think of any goals you want to achieve? Remember that your goals don't have to be big – they can be something as simple as washing the dog on the weekend.

If you are thinking, "What's the point of setting goals?", remember that just by accomplishing something, you will feel better.

Commit

Commit to setting yourself a small goal and rewarding yourself when you reach it. Right now, set aside a minute to plan a goal you would like to achieve this week.

Imagine **when** and **where** you are going to do it. Write your goal down, or remember to tell someone your goal. You can also print this email as a reminder.

Example

"*This Saturday morning I will wash the dog myself so that he doesn't dirty the floors. I will buy myself a magazine as a reward.*"

Past commitments

In the last Mood Memo we sent you, we encouraged you to **fight the desire to do nothing**. *How did you go?*

© 2010 – 2011 MoodMemos.com | Orygen Youth Health | University of Melbourne | 35 Poplar Road Parkville VIC 3052 Australia

Amy you have received this email because you signed up to take part in the MoodMemos.com study. If you want to stop receiving emails from us and withdraw from the study, please unsubscribe here.

Having trouble reading this email? View it in a web browser

mood memos

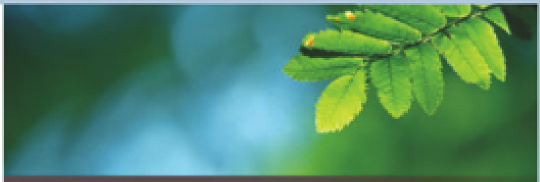

#4 Eat well

Dear Amy,

You can help yourself feel better by **eating a healthy, balanced diet**.

Why?

- Eating a healthy, balanced diet is hard when you are depressed because you tend to neglect to care for yourself.
- Some people find they lose their appetite and don't want to eat, but not eating makes you feel run down.
- Others draw comfort from eating sweet or fatty foods, but this can make you feel worse because of guilty feelings, and may cause weight gain resulting in fatigue.
- Eating a healthy diet will give your body the nutrients it needs to improve your overall vitality and ability to concentrate, as well as reducing irritability and risk of illness.

How?

- Eat a variety of foods every day.
- Make sure to include lots of fruits and vegetables, and eat less fatty, salty, sugary foods.
- More healthy eating tips can be found at the Australian Guide to Healthy Eating
- **If you are overeating for comfort**, think about the high risk times when you often overeat (e.g. in the afternoon, or after a stressful experience). Plan what you will do during those times (e.g. eat some fruit instead, or go for a walk).

But...

What if you are eating healthily but then slip up and give in to your cravings? Try not to beat yourself up about it. The occasional slip is ok.

What if you want to eat healthily but don't feel hungry and forget to eat? Try to eat at regular times. Setting an alarm or leaving a message lying around reminding yourself to eat can be helpful.

What if you don't enjoy food and nothing tastes good anymore? It may help to eat with others, or eat foods you would normally enjoy.

Commit

Make a commitment to eat a healthy, balanced diet. Right now, set aside a minute to plan a goal you would like to achieve this week.

Imagine **when** and **where** you are going to do it. Write your goal down, or remember to tell someone your goal. You can also print this email as a reminder.

Example

“*This week I will pack some fruit each day to eat in the afternoon when I get cravings.*”

Past commitments

In the last Mood Memo we sent you, we encouraged you to **set yourself a small goal and reward yourself when you reach it**. *How did you go?*

© 2010 – 2011 MoodMemos.com | Orygen Youth Health | University of Melbourne | 35 Poplar Road Parkville VIC 3052 Australia

Amy you have received this email because you signed up to take part in the MoodMemos.com study. If you want to stop receiving emails from us and withdraw from the study, please unsubscribe here.

Having trouble reading this email? View it in a web browser

mood memos

#5 Improve your sleep habits

Dear Amy,

If you are feeling a bit depressed, one way to feel better is by **improving your sleep habits**.

Why?

- Poor sleep plays a role in depression.
- Poor sleep makes you feel exhausted, more stressed, and harder to think clearly.
- Improving your sleep habits was recommended by more than 80% of therapists and people who have recovered from depression.

How?

- Go to bed and get up at the same time each day (even the weekend).
- Wind down with relaxing activities before bedtime.
- Don't drink alcohol in the evening – it can cause you to wake up in the middle of the night.
- When you can't sleep, get out of bed and do something in another room. Go back to bed when you're feeling drowsy.
- Don't read or watch TV in bed. Only use your bed for sleep and sex.
- Keep your bedroom quiet and a comfortable temperature.
- Don't nap during the day no matter how tired you feel.

But...

What if you don't want to have a fixed sleep routine but prefer being spontaneous? Just remember that if you persist in following the advice above, your new sleep habits will become natural for you.

What if you have followed the advice above, but you still wake up feeling tired after a poor night's sleep? Just remember that improving your sleep is a gradual process and try to persist with your good habits.

What if your sleep routine changes a lot because you work shifts? You can still apply most of the tips above to improve your sleep habits.

Commit

Commit to improve your sleep habits. Right now, set aside a minute to plan a goal you would like to achieve this week.

Imagine **when** and **where** you are going to do it. Write your goal down, or remember to tell someone your goal. You can also print this email as a reminder.

Example

"*This week I will try to go to bed at 11pm each night, and get up at 7am each morning.*"

Past commitments

In the last Mood Memo we sent you, we encouraged you to **eat a healthy, balanced diet**. *How did you go?*

© 2010 – 2011 MoodMemos.com | Orygen Youth Health | University of Melbourne | 35 Poplar Road Parkville VIC 3052 Australia

Amy you have received this email because you signed up to take part in the MoodMemos.com study. If you want to stop receiving emails from us and withdraw from the study, please unsubscribe here.

mood memos

#6 Do something you enjoy that gives you a sense of achievement

Dear Amy,

If you are feeling a bit depressed, one way to feel less depressed is to **do something you enjoy and that gives you a sense of achievement**.

Why?

- Depression interferes with your ability to feel positive emotion and leads to doing fewer enjoyable activities.
- Doing activities that are enjoyable and that give you a sense of achievement provides you with more opportunities to feel good about yourself and your life.
- Doing more activities that are enjoyable and that give a sense of achievement was recommended by more than 80% of therapists and people who have recovered from depression.

How?

- Think about the activities you usually enjoy doing. Have you stopped doing these lately? Maybe it is time to start again.
- Brainstorm a list of things you enjoy or used to enjoy doing
- This can be simple things like surfing interesting videos on YouTube, watching a funny movie, going out to a favourite restaurant, having a luxurious bath, playing with your pet, or reading a good book.
- Even better are activities that take more effort and give you a sense of accomplishment, such as learning something new, doing some simple DIY around the house, planning a holiday or upcoming birthday, trying a recipe you have never cooked before, or doing a small task that you have been putting off.

But...

You might be thinking that there is nothing enjoyable that you want to do. That's ok – that's part of the depression. Why not try asking a friend or family member to help you think of some things you can do?

Remember that you might not find your activities enjoyable at first. All you can do is make a small start and gradually your enjoyment will increase over time.

Commit

Make a commitment to do more enjoyable activities. Right now, set aside a minute to plan a goal you would like to achieve this week.

Imagine **when** and **where** you are going to do it. Write your goal down, or remember to tell someone your goal. You can also print this email as a reminder.

Example

“*This week I will visit the video store and hire two comedy DVDs, which I will watch in the evening after dinner.*”

Past commitments

In the last Mood Memo we sent you, we encouraged you to commit to **improve your sleep habits**. *How did you go?*

© 2010 – 2011 MoodMemos.com | Orygen Youth Health | University of Melbourne | 35 Poplar Road Parkville VIC 3052 Australia

Amy you have received this email because you signed up to take part in the MoodMemos.com study. If you want to stop receiving emails from us and withdraw from the study, please unsubscribe here.

mood memos

#7 Talk to someone supportive about your problems and how you feel

Dear Amy,

If you want to feel less depressed, why not try **talking to someone who is supportive and caring about your problems or how you feel?**

Why?

- Talking about your problems with someone can help you feel supported and less alone.
- It helps to talk about what you are going through rather than holding things in.
- Other people often have a different perspective on your problems that could be helpful.
- Talking about your problems and feelings with someone who is supportive and caring was recommended by more than 80% of therapists and people who have recovered from depression.

How?

- Think about someone you trust who cares about you
- Arrange to talk to them about how you are feeling and what problems are happening in your life
- Let them know that you appreciate their support

But...

What if you can't think of anyone you want to talk to, or you have tried in the past and they were not supportive? Another option is trying a depression forum on the Internet, where sharing is encouraged.

Do your problems seem too big to be helped by talking to someone? That's ok – just talking about how you feel can make you feel better, even if there are no easy solutions.

What if you don't want to burden anyone with your problems? Remember that it can also make the other person feel wanted and trusted because you have confided in them.

Commit

Make a commitment to talk to someone supportive about your problems and how you feel. Right now, set aside a minute to plan how you will do this.

Imagine **when** and **where** you are going to do it. Write your goal down, or remember to tell someone your goal. You can also print this email as a reminder.

Example

“*I will talk to my sister about how I am going. I will call her tonight at 8pm, and I will leave her a message to call me back if she is not in.*”

Past commitments

In the last Mood Memo we sent you, we encouraged you to commit to **doing more enjoyable activities**. *How did you go?*

© 2010 – 2011 MoodMemos.com | Orygen Youth Health | University of Melbourne | 35 Poplar Road Parkville VIC 3052 Australia

Amy you have received this email because you signed up to take part in the MoodMemos.com study. If you want to stop receiving emails from us and withdraw from the study, please unsubscribe here.

Having trouble reading this email? View it in a web browser

mood memos

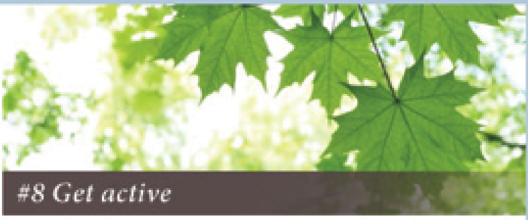

Dear Amy,

You can help yourself feel less depressed by **doing regular exercise or physical activity.**

Why?

- Scientific studies have shown that regular moderate exercise is an effective treatment for depression.
- Not only will you improve your physical health, but exercise will improve your mood, decrease anxiety, and make you feel better about yourself.
- Getting active by exercising was recommended by more than 80% of therapists and people who have recovered from depression.

How?

- Choose an activity you enjoy and start with small, flexible goals.
- Getting active can be as simple as doing the vacuuming, taking the stairs instead of the lift, doing some gardening, going for a walk in the park, washing and vacuuming your car, or walking/riding/skating to get where you're going.
- Getting active could also be an extra activity that you take up or schedule each day, such as going for a brisk walk at lunch time, taking up a team sport, going for a jog after work, learning to play golf, or taking dance or yoga classes.
- Exercising with others can be motivating and fun. Alternatively, if you need relief from the pressures and demands of others, choose a solitary activity.

But...

You might be thinking you know exercise is good for you but you don't have the energy and it sounds too hard. The key is to start with something **small**, such as doing a little more activity than what you are doing now. Aim to build it up over time as your energy and mood improve.

Commit

Make a commitment to do regular exercise or physical activity. Right now, set aside a minute to plan a goal you would like to achieve this week.

Imagine **when** and **where** you are going to do it. Write your goal down, or remember to tell someone your goal. You can also print this email as a reminder.

Example

(If you are not doing any physical activity at all): “*I will aim to go for a 15-minute walk in the park twice this week, during my lunch break.*”

Past commitments

In the last Mood Memo we sent you, we encouraged you to commit to **talk to someone supportive about your problems and how you feel**. *How did you go?*

© 2010 – 2011 MoodMemos.com | Orygen Youth Health | University of Melbourne | 35 Poplar Road Parkville VIC 3052 Australia

Amy you have received this email because you signed up to take part in the MoodMemos.com study. If you want to stop receiving emails from us and withdraw from the study, please unsubscribe here.

Having trouble reading this email? View it in a web browser

mood memos

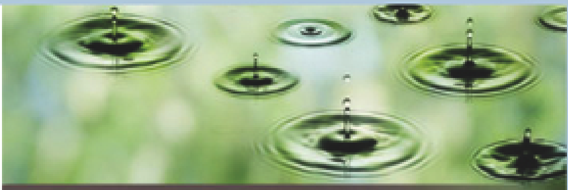

#9 Do what has worked for you in the past

Dear Amy,

If you are feeling a bit depressed, one way to feel less depressed is to **do things that have worked for you in the past**.

Why?

- You might have forgotten that there are things you can do to cheer yourself up or make you feel less depressed.
- You will know what things work best for you. If they worked in the past, they will probably be helpful now.
- Doing things that have worked in the past was recommended by more than 80% of therapists and people who have recovered from depression.

How?

- Try to think of other times when you were depressed or felt sad or down.
- What things did you do during those times that you found helpful?
- Make a list of these things and think about how you can do them now

But...

What if you haven't been depressed before? You could go back over the previous mood memos and focus on the ones that seem the best for you.

What if you were depressed in the past but nothing you did helped? Your situation could be different now – it might be worth trying them again. Or alternatively, you could read over the previous mood memos and focus on the ones that seem the best for you.

Commit

Make a commitment to write a list of strategies that have worked in the past for you and use them. Take time now to think of strategies, and then plan when and where during the week you will do them.

Write your goal down, or remember to tell someone your goal. You can also print this email as a reminder.

Example

“*In the past I have felt better after getting out in the morning sunshine. This week, I will spend 15 minutes outside in the sunshine on two mornings.*”

Past commitments

In the last Mood Memo we sent you, we encouraged you to commit to **do regular exercise or physical activity**. *How did you go?*

© 2010 – 2011 MoodMemos.com | Orygen Youth Health | University of Melbourne | 35 Poplar Road Parkville VIC 3052 Australia

Amy you have received this email because you signed up to take part in the MoodMemos.com study. If you want to stop receiving emails from us and withdraw from the study, please unsubscribe here.

Having trouble reading this email? View it in a web browser

mood memos

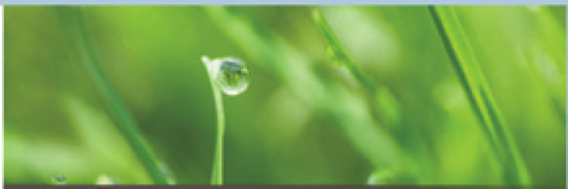

#10 Let others know how you are feeling

Dear Amy,

You can help yourself feel less depressed by **letting friends and family know how you are feeling so they know what you are going through**.

Why?

- When you tell them something is wrong, you give them the opportunity to support you.
- Telling them how you are feeling lets them understand why you might not have been yourself lately.
- Letting friends and family know how you are feeling was recommended by more than 80% of therapists and people who have recovered from depression.

How?

- Think about the important people in your life, who may have noticed that you are not feeling right, or are acting differently.
- These could be family members, close friends, co-workers, or your boss.
- Talk with each of them to let them know how you have been feeling lately.

But...

You might be thinking that your family and friends don't want to know if you are not doing well and you should put on a brave face. But it is important to be honest with them, even just to let them know that they are not at fault.

What if you are afraid to tell them in case they think less of you? That's ok – this negative thought is probably part of your depression, and they won't think this.

Commit

Make a commitment to tell a friend or family member how you are feeling. Right now, set aside a minute to plan how you will achieve this.

Imagine **when** and **where** you are going to do it. Write your goal down, or remember to tell someone your goal. You can also print this email as a reminder.

Example

“*Tomorrow morning at work I will talk to a close colleague about how I have been feeling.*”

Past commitments

In the last Mood Memo we sent you, we encouraged you to **write a list of strategies that have worked in the past for you and use them.** *How did you go?*

© 2010 – 2011 MoodMemos.com | Orygen Youth Health | University of Melbourne | 35 Poplar Road Parkville VIC 3052 Australia

Amy you have received this email because you signed up to take part in the MoodMemos.com study. If you want to stop receiving emails from us and withdraw from the study, please unsubscribe here.

mood memos

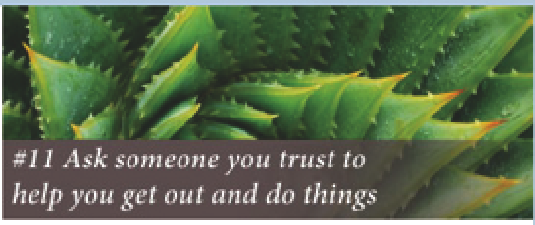

Dear Amy,

If you are feeling a bit depressed, one way to feel less depressed is to **ask a trusted friend or relative to help you get out and about or do activities**.

Why?

- Sometimes it can be hard to find the energy and motivation to do things on your own when you feel depressed.
- Having a friend or relative help you means you are more likely to get out and about which will improve your mood.
- Asking a trusted friend or relative to help you get out and about or do activities was recommended by more than 80% of therapists and people who have recovered from depression.

How?

- Think about someone you trust who will be able to support you.
- Think about things they could help you do, such as specific activities you need motivation to do, or just making sure you get out of the house each day.
- Ask them if they would be willing to help you in this way.
- Plan with them how they will help you, for example, will they meet up with you and do activities, or will they phone you regularly to check what you have been up to?

But...

What if you can't think of anyone you trust to help you in this way? You could join an activity group that meets regularly. For example, a support group, an exercise or sporting group, or simply meet someone regularly for lunch. Having a regular arrangement to meet other people for an activity is the important thing.

What if you think you would be imposing on people? Remember that people who care about you want you to get better and will welcome the opportunity to be of some help.

Commit

Make a commitment to ask someone you trust to help you get out and about or do activities. Right now, set aside a minute to think about who you will ask and how they might help you.

Set a goal for when and where you are going to do it. Write your goal down, or remember to tell someone your goal. You can also print this email as a reminder.

Example

“*I will ask my sister if she would like to go see a movie with me this weekend. I will call her tonight at 8pm to discuss it, and I will leave her a message to call me back if she is not in.*”

Past commitments

In the last Mood Memo we sent you, we encouraged you to **tell a friend or family member how you are feeling**. *How did you go?*

© 2010 – 2011 MoodMemos.com | Orygen Youth Health | University of Melbourne | 35 Poplar Road Parkville VIC 3052 Australia

Amy you have received this email because you signed up to take part in the MoodMemos.com study. If you want to stop receiving emails from us and withdraw from the study, please unsubscribe here.

mood memos

#12 Learn relaxation techniques

Dear Amy,

If you want to feel less depressed, what about **learning relaxation techniques such as progressive muscle relaxation, autogenic training, breathing exercises, or even self-hypnosis?**

Why?

- Too much stress can leave you with headaches, low mood, poor sleep, and feeling tense and irritable.
- Practising relaxation techniques can stop the effects of stress on your body, leaving you less anxious and feeling less depressed.
- Scientific studies have shown that learning relaxation techniques is helpful for depression.
- Relaxation techniques are a useful skill that you can re-use in the future when you feel stressed.

How?

- You can teach yourself these techniques by reading books or listening to audio CDs or mp3s that teach the techniques.
- Progressive muscle relaxation teaches you to tense and then release muscle groups in your body.
- Autogenic training teaches you to concentrate on your breathing, heartbeat, and the warmth and heaviness of parts of your body.
- These techniques are best practised in a quiet place where you feel comfortable and have some privacy.
- Learning to relax is a skill, and like other skills such as playing the piano, regular practice is best.

But...

Can't find the time to practise relaxation? Some techniques only require a few minutes practice. Try incorporating the practice into your daily routine, such as after you wake up, or before you go to bed.

What if you've tried it but had strange feelings, such as tingling or a floating sensation? This is a good sign that the muscles are beginning to loosen. The key is to *go with it* and be free of any worry about how well you are performing the technique.

What if you feel that you are already relaxed? These relaxation techniques are worth learning because they produce a deeper type of relaxation than everyday relaxation.

Commit

Make a commitment to learn relaxation techniques. Right now, set aside a minute to plan a goal you would like to achieve this week.

Imagine **when** and **where** you are going to do it. Write your goal down, or remember to tell someone your goal. You can also print this email as a reminder.

Example

“*I will download a relaxation mp3 from the web today. I will listen to it at least 3 nights this week before I go to bed.*”

Past commitments

In the last Mood Memo we sent you, we encouraged you to **ask someone you trust to help you get out and about or do activities**. *How did you go?*

© 2010 – 2011 MoodMemos.com | Orygen Youth Health | University of Melbourne | 35 Poplar Road Parkville VIC 3052 Australia

Amy you have received this email because you signed up to take part in the MoodMemos.com study. If you want to stop receiving emails from us and withdraw from the study, please unsubscribe here.
