# Supplementary material for: Self-Help for Depression via E-mail: A Randomised Controlled Trial of Effects on Depression and Self-Help Behaviour
Source: PLoS One. 2013 Jun 21;8(6):e66537. doi: 10.1371/journal.pone.0066537 (PMC3689826; doi:10.1371/journal.pone.0066537)
Supplement: Control emails S1 — Screenshots of the control group emails. (PDF) [file pone.0066537.s004.pdf]

Having trouble reading this email? View it in a web browser

mood memos

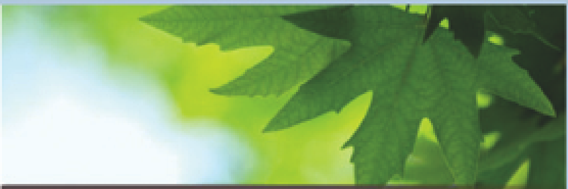

#1 What is depression?

Dear Amy,

Myth

"Depression is just having a low mood."

The facts

Major depression is a **serious health problem**. It is more than just a low mood and makes it hard for people to function every day.

Want to know more?

Because there is no biological test to diagnose major depression, it is defined by its symptoms, which must have lasted for **at least two weeks**.

Symptoms affect emotions, thoughts, behaviour and physical health.

Emotions

- Feeling sad or miserable most of the time
- Feeling guilty, worthless, anxious, hopeless

Thoughts

- Poor memory and concentration
- Thoughts of death or suicide
- Negative thinking about the self, world, and future

Behaviour

- Loss of motivation
- Loss of interest in or enjoyment from usual activities
- Withdrawal from others

Physical

- Feeling tired all the time
- Sleep problems
- Changes in appetite or weight
- Unexplained aches and pains
- Moving more slowly **or** being agitated and unable to settle

© 2010 – 2011 MoodMemos.com | Orygen Youth Health | University of Melbourne | 35 Poplar Road Parkville VIC 3052 Australia

Amy you have received this email because you signed up to take part in the MoodMemos.com study. If you want to stop receiving emails from us and withdraw from the study, please unsubscribe here.

Having trouble reading this email? View it in a web browser

mood memos

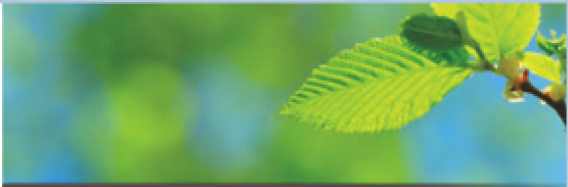

#2 What types of depression are there?

Dear Amy,

Myth

"The experience of depression is the same for everybody."

The facts

As well as **major depression**, there are several other types of depression that have different symptom patterns. Some examples are below.

Want to know more?

Psychotic depression

- This is a more **severe form** of depression where people have delusions or hallucinations
- It is experienced by about 10–15% of people with depression
- It more often occurs in people **over 50 years**

Seasonal affective disorder

- This occurs in **specific seasons**, usually autumn/fall or winter, with recovery in spring
- It is more common **further away from the equator** where there is less sunlight during winter
- Features fatigue, and a tendency to crave sugars, overeat, and oversleep

Atypical depression

- It often begins earlier in life and is more common in females and in people with other mental health problems
- **Sleeping and eating too much** is more common than insomnia and a loss of appetite
- Other features are a heavy, leaden feeling in arms or legs, being extra sensitive to social rejection, and mood improving in response to positive events

© 2010 – 2011 MoodMemos.com | Orygen Youth Health | University of Melbourne | 35 Poplar Road Parkville VIC 3052 Australia

Amy you have received this email because you signed up to take part in the MoodMemos.com study. If you want to stop receiving emails from us and withdraw from the study, please unsubscribe here.

Having trouble reading this email? View it in a web browser

mood memos

#3 Can depression cause physical health problems?

Dear Amy,

Myth

"Depression is all in the mind so it is unrelated to physical health problems."

The facts

Depression is strongly linked with many physical health problems, such as heart disease, diabetes, asthma and cancer.

Want to know more?

- Rates of depression are higher in people with chronic health problems, such as asthma.
- This could be due to the stress from having the illness, the drugs used to treat it, or they could share common biological causes.
- Having depression also increases your risk of having health problems too.
- Depression **increases the risk of having heart disease by 60%**, and is also linked with poorer recovery.
- Depression also increases your risk of having **type-2 diabetes by more than 30%**.

© 2010 - 2011 MoodMemos.com | Orygen Youth Health | University of Melbourne | 35 Poplar Road Parkville VIC 3052 Australia

Amy you have received this email because you signed up to take part in the MoodMemos.com study. If you want to stop receiving emails from us and withdraw from the study, please unsubscribe here.

Having trouble reading this email? View it in a web browser

mood memos

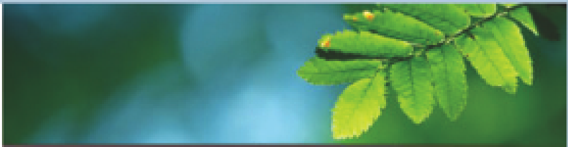

#4 Depression & other mental health problems

Dear Amy,

Myth

"People with depression usually don't have other mental health problems."

The facts

Many people with depression will also experience another mental health problem during their lifetime.

Want to know more?

- **Anxiety disorders** are very common — about **50%** of people with depression also have an anxiety disorder (such as panic disorder or social anxiety disorder).
- This could be because the risks for having depression and anxiety are similar (e.g. childhood upbringing).
- Substance use problems, such as alcohol abuse, are also common in people with depression.
- For some people, alcohol abuse is the cause of their depression, and their depression improves when they stop drinking.

© 2010 – 2011 MoodMemos.com | Orygen Youth Health | University of Melbourne | 35 Poplar Road Parkville VIC 3052 Australia

Amy you have received this email because you signed up to take part in the MoodMemos.com study. If you want to stop receiving emails from us and withdraw from the study, please unsubscribe here.

Having trouble reading this email? View it in a web browser

mood memos

#5 What is the history of depression?

Dear Amy,

Myth

"Depression is a modern disease."

The facts

Depression was described by the **Ancient Greeks 2,500 years ago**, when it was known as melancholia.

Want to know more?

- Hippocrates described it as an *aversion to food, despondency, sleeplessness, irritability, and restlessness.*
- The Ancient Greeks thought diseases were caused by an excess in the body of one of four elements: black bile, yellow bile, phlegm, or blood. **Melancholia** was thought to be caused by too much **black bile**.
- The Romans also believed black bile caused melancholia, specifically when too much built up below the rib cage.
- This idea lasted for thousands of years, but began to change near the end of the 18th century (late 1700s), when **life circumstances** were thought to play a role.
- By the end of the 19th century (1800s) the term depression was used as much as melancholia.

© 2010 – 2011 MoodMemos.com | Orygen Youth Health | University of Melbourne | 35 Poplar Road Parkville VIC 3052 Australia

Amy you have received this email because you signed up to take part in the MoodMemos.com study. If you want to stop receiving emails from us and withdraw from the study, please unsubscribe here.

Having trouble reading this email? View it in a web browser

mood memos

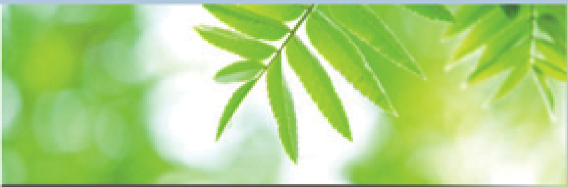

#6 What causes depression?

Dear Amy,

Myth

"Depression is only caused by stressful life experiences."

The facts

Depression doesn't have a single cause — **both genetics and environment play a role** in causing depression.

Want to know more?

- Environmental causes include childhood upbringing (such as child abuse or neglect), and stressful life experiences (such as the death of a loved one, unemployment, being in an abusive relationship).
- Genetic makeup explains about **40% of the risk** of having depression.
- Environmental causes interact with genetics to cause depression.

How do environmental experiences and genetics interact to cause depression?

Sensitivity to stress

- One example is through sensitivity to stress, which is partly genetic.
- High sensitivity to stress combined with stressful life events increases the risk of developing depression.

Personality

- Another example is through personality, which is partly genetic.
- There is a personality trait that involves having more negative emotions and difficulty controlling emotions.
- This personality trait increases the risk of having stressful life experiences.
- It is also increases the risk of having depression.

© 2010 – 2011 MoodMemos.com | Orygen Youth Health | University of Melbourne | 35 Poplar Road Parkville VIC 3052 Australia

Amy you have received this email because you signed up to take part in the MoodMemos.com study. If you want to stop receiving emails from us and withdraw from the study, please unsubscribe here.

Having trouble reading this email? View it in a web browser

mood memos

#7 Who is at risk of depression?

Dear Amy,

Myth

"Everyone is equally at risk of having depression."

The facts

Some people are **more at risk** of having depression than others.

Want to know more?

- Depression is **twice as common in women** than in men. This may be because of differences in hormones, social roles, coping skills, or exposure to abuse and stressful life experiences.
- This difference only emerges **after adolescence** — before then rates of depression are similar between boys and girls.

Others more at risk are people who:

- are not married
- have a low income and level of education
- experienced early childhood trauma (e.g. abuse or neglect)
- have relatives with depression
- have experienced stressful life events, especially those involving loss (e.g. unemployment, divorce)

© 2010 – 2011 MoodMemos.com | Orygen Youth Health | University of Melbourne | 35 Poplar Road Parkville VIC 3052 Australia

Amy you have received this email because you signed up to take part in the MoodMemos.com study. If you want to stop receiving emails from us and withdraw from the study, please unsubscribe here.

Having trouble reading this email? View it in a web browser

mood memos

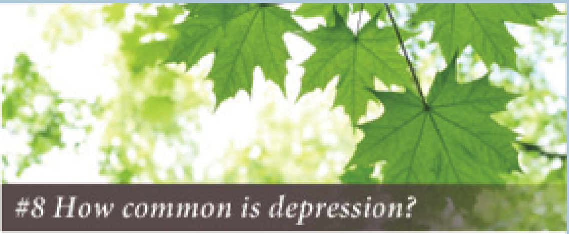

Dear Amy,

Myth

"Depression is not a common problem."

The facts

Depression is one of the **most common mental health problems**.

Want to know more?

- Rates vary across countries, but in Western societies such as the USA, about **16%** of people will experience depression at some point in their lives, and about **6% during any year**.
- Depression may be less common in Asian societies.

Is depression becoming more common?

- Rates of depression have been studied for 3 decades, and there seems to be an increase in depression over time.
- It may be that depression is more common in people born more recently.
- Or it may just appear to be more common because people feel more comfortable reporting it, or because tools to measure depression are more accurate.

© 2010 – 2011 MoodMemos.com | Orygen Youth Health | University of Melbourne | 35 Poplar Road Parkville VIC 3052 Australia

Amy you have received this email because you signed up to take part in the MoodMemos.com study. If you want to stop receiving emails from us and withdraw from the study, please unsubscribe here.

Having trouble reading this email? View it in a web browser

mood memos

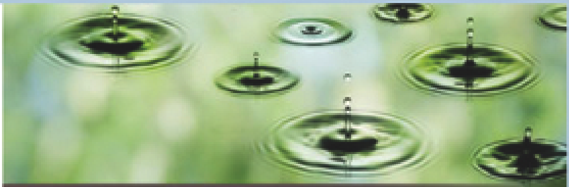

#9 Burden of depression

Dear Amy,

Myth

"Depression is a personal problem and does not affect the wider community."

The facts

When a person has depression **the community suffers as well**. This is because depression reduces a person's ability to function well at work, school, and in the home, and it affects relationships with family and friends.

Want to know more?

Depression is a big problem in the community.

- Depression causes **more disability during the middle years of life** than any other health problem.
- In fact, the World Health Organization ranks depression as the **4th most urgent health problem worldwide**.
- The cost of depression to the community is huge.
- Depression cost the USA an estimated $53 billion dollars in 1996.
- These costs are from people not being able to work, not working productively when at work, treatment costs, and costs due to suicide.

© 2010 – 2011 MoodMemos.com | Orygen Youth Health | University of Melbourne | 35 Poplar Road Parkville VIC 3052 Australia

Amy you have received this email because you signed up to take part in the MoodMemos.com study. If you want to stop receiving emails from us and withdraw from the study, please unsubscribe here.

Having trouble reading this email? View it in a web browser

mood memos

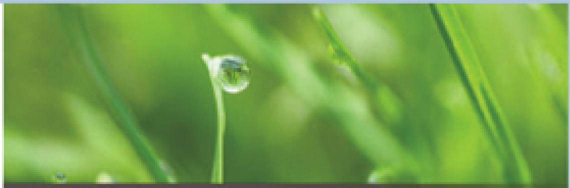

#10 Is depression a weakness?

Dear Amy,

Myth

"Only the weak get depression."

The facts

This negative belief is **just not true**. Depression affects all kinds of people, and is not a barrier to success in life.

Want to know more?

Here are just some well-known successful people who have had depression:

- Winston Churchill – prime minister of the UK during WWII
- Abraham Lincoln – 16th president of the United States
- Charles Dickens – author of David Copperfield and Oliver Twist
- T.S. Eliot – Nobel prize for literature winner
- Diana, Princess of Wales
- Jim Carey – Canadian actor and comedian
- JK Rowling – British author of the Harry Potter books
- Marcus Trescothick – English cricketer
- Nathan Thompson – Australian football player

The good news is that community campaigns are slowly improving people's negative beliefs about depression.

One of the best ways to improve negative beliefs is to have contact with someone who has had depression.

© 2010 – 2011 MoodMemos.com | Orygen Youth Health | University of Melbourne | 35 Poplar Road Parkville VIC 3052 Australia

Amy you have received this email because you signed up to take part in the MoodMemos.com study. If you want to stop receiving emails from us and withdraw from the study, please unsubscribe here.

Having trouble reading this email? View it in a web browser

mood memos

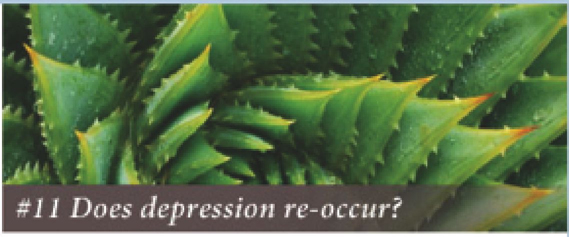

Dear Amy,

Myth

"Once a person recovers from depression they are unlikely to have it again during their lifetime."

The facts

Depression is a recurring problem for many people. Depression occurs in **episodes** and people who get depression are likely to have another episode during their lifetime.

Want to know more?

- Each episode of depression lasts for **6 months** on average.
- Up to **3 out of 4** people will have another episode after recovering.
- The average number of episodes over a lifetime is 5 – 6, or about one every 5 years.

What increases the risk of having depression more than once?

- Having a family history of depression
- Having another mental health problem before depression began
- Having depression for the first time at a young age

© 2010 – 2011 MoodMemos.com | Orygen Youth Health | University of Melbourne | 35 Poplar Road Parkville VIC 3052 Australia

Amy you have received this email because you signed up to take part in the MoodMemos.com study. If you want to stop receiving emails from us and withdraw from the study, please unsubscribe here.

Having trouble reading this email? View it in a web browser

mood memos

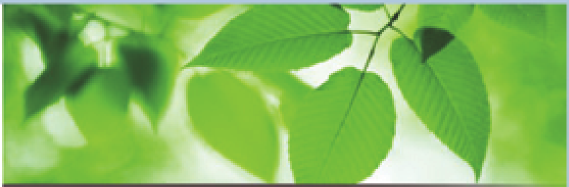

#12 Depression across cultures

Dear Amy,

Myth

"Depression only occurs in modern Western societies."

The facts

Depression can be identified in **all cultures**.

Want to know more?

- Although depression is universal, it varies in how common it is, and how its symptoms are described.
- This is because different cultures have particular ways of talking about distress, that are easily understood by others in that culture.

How is depression described in other cultures?

- A common way of describing depression around the world is as a **soul loss** or **spirit loss**.
- In Chinese culture, people are more likely to talk about physical symptoms (such as pain) than to report feeling depressed. This may be because talking about mood is seen as self-centred and a threat to social ties.
- In the Caribbean, depression is described as pressure rising in the head.
- In equatorial Africa, it is described as sensations of heat or peppery feeling in the head.
- In Iran, it is described as a heavy heart or chest tightness.

© 2010 – 2011 MoodMemos.com | Orygen Youth Health | University of Melbourne | 35 Poplar Road Parkville VIC 3052 Australia

Amy you have received this email because you signed up to take part in the MoodMemos.com study. If you want to stop receiving emails from us and withdraw from the study, please unsubscribe here.
